# Impact of BAFF Blockade on Inflammation, Germinal Center Reaction and Effector B-Cells During Acute SIV Infection

**DOI:** 10.3389/fimmu.2020.00252

**Published:** 2020-02-28

**Authors:** Gwenoline Borhis, Maria Trovato, Hany M. Ibrahim, Stephane Isnard, Roger Le Grand, Nathalie Bosquet, Yolande Richard

**Affiliations:** ^1^Université de Paris, Institut Cochin, INSERM, CNRS, Paris, France; ^2^CEA, Université Paris Sud, INSERM U1184, Immunology of Viral Infections and Autoimmune Diseases (IMVA), IDMIT Department/IBFJ, Fontenay-aux-Roses, France

**Keywords:** B-cells, BAFF, GC, T_FH_, memory B-cells, SIV, CXCL13, pDC

## Abstract

Memory B-cell dysfunctions and inefficient antibody response suggest germinal center (GC) impairments during HIV/SIV infection with possible contribution of overproduced B-cell activating factor (BAFF). To address this question, we compared proportions and functions of various B-cell subsets and follicular helper T-cells (T_FH_) in untreated (Placebo) and BR3-Fc treated (Treated) SIV-infected macaques. From day 2 post-infection (dpi), Treated macaques received one weekly injection of BR3-Fc molecule, a soluble BAFF antagonist, for 4 weeks. Whereas, the kinetics of CD4^+^ T-cell loss and plasma viral loads were comparable in both groups, BAFF blockade delayed the peak of inflammatory cytokines (CXCL10, IFNα), impaired the renewal of plasmacytoid dendritic cells and fostered the decline of plasma CXCL13 titers after 14 dpi. In Treated macaques, proportions of total and naïve B-cells were reduced in blood and spleen whereas SIV-induced loss of marginal zone (MZ) B-cells was only accentuated in blood and terminal ileum. Proportions of spleen GC B-cells and T_FH_ were similar in both groups, with CD8^+^ T-cells and rare Foxp3^+^ being present in spleen GC. Regardless of treatment, sorted T_FH_ produced similar levels of IL21, CXCL13, and IFNγ but no IL2, IL4, or BAFF and exhibited similar capacities to support IgG production by autologous or heterologous B-cells. Consistently, most T_FH_ were negative for BAFF-R and TACI. Higher proportions of resting and atypical (CD21^lo^) memory B-cells were present in Treated macaques compared to Placebo. In both groups, we found higher levels of BAFF-R expression on MZ and resting memory B-cells but low levels on atypical memory B-cells. TACI was present on 20-30% of MZ, resting and atypical memory B-cells in Placebo macaques. BAFF blockade decreased TACI expression on these B-cell subsets as well as titers of SIV-specific and vaccine-specific antibodies arguing for BAFF being mandatory for plasma cell survival. Irrespective of treatment, GC B-cells expressed BAFF-R at low level and were negative for TACI. In addition to key information on spleen BAFF-R and TACI expression, our data argue for BAFF contributing to the GC reaction in terminal ileum but being dispensable for the generation of atypical memory B-cells and GC reaction in spleen during T-dependent response against SIV.

## Introduction

Optimal protection against numerous pathogens requires humoral memory response with the rapid development of neutralizing antibodies (Abs) and the generation of pathogen-specific effector B cells (long-lived plasma blasts and memory B-cells) in germinal centers (GC). Whereas, GC hyperplasia is the first sign of ongoing B-cell response described in HIV-infected patients (1), HIV-specific Ab production is globally inefficient in containing virus replication and in preventing the establishment of viral reservoirs (2, 3). Cross-neutralizing Abs with narrow breadth are present during the 1st year of infection in high numbers of HIV-infected individuals (4), but only a minority of individuals develops potent broadly neutralizing Abs after years of infection. These Abs generally harbor features of self/poly-reactivity and high levels of somatic hyper-mutations in immunoglobulin (Ig) genes, arguing for their T-dependent (TD) affinity maturation and selection in GC (5–7). Emergence and breadth of such Abs rely on a dynamic combination of factors including viral load and fitness, CD4 T-cell counts, inflammation and B-cell changes. In particular, increased proportions of atypical (CD21^lo^) memory B-cells and loss in conventional, Resting Memory (RM, CD21^+^CD27^+^) B-cells are hallmark features of chronic infection by HIV or Simian Immunodeficiency virus (SIV) (8). Among atypical memory B-cells, two subsets have been characterized: Tissue-like memory B-cells (TLM, CD21^lo^CD27^lo^) enriched in Envelope-specific B-cells (9, 10) and Activated Memory B-cells (AM, CD21^lo^CD27^+^). Rapid loss in AM B-cells at the acute phase of infection predicts accelerated progression toward the chronic phase (11). First considered as exhausted B-cells due to the expression of FcRL5/4 and PD1 (12), TLM B-cells are now thought to be short-lived, early plasma blasts or recent GC emigrants that are maintained by repetitive antigen exposure during chronic infection but also physiologically during vaccination (13). Both TLM and AM highly express FcRL4 and overproduce IL6 in chronically HIV-infected individuals, with FcRL4^hi^IL6^hi^ AM acting as potent pro-inflammatory B-cells (14). During chronic infection, TLM B-cells produce poly-reactive IgG, part of them being protective/neutralizing Abs. Functional HIV-specific memory B-cells are associated with efficient viral neutralization in Elite controllers (10, 15), and significant recovery of HIV-specific RM B-cells requires initiation of anti-retroviral therapy as soon as the acute phase of infection (16).

HIV-specific memory B-cells and plasma blasts are generated during a multistep process in GC (17), thus identification of key impairments occurring in GC during natural infection would provide important clues to improve their generation and efficiency in terms of neutralization. Mandatory for B-cell selection in GC, follicular helper T-cells (T_FH_) are present at elevated frequencies in GC during HIV/SIV infection but frequently exhibit impaired helper functions and cytokine production during the chronic phase (18–22) and possibly during the acute phase (23). Preserved helper functions during early phases of infection are determinant for the development of broadly neutralizing Abs at the chronic phase (24–26). Elevated level of circulating CXCL13, thought to be a reliable marker of GC activity during vaccination (27), was shown to predict the emergence of early cross-neutralizing HIV Abs (28).

Whereas, BAFF (B-cell-activating factor belonging to the tumor necrosis factor superfamily) and its receptors BAFF-R/BR3 (BLys receptor 3) and TACI (transmembrane activator and CAML interactor) are dispensable for GC initiation, physiological levels of BAFF are optimal for the development of high-affinity Abs (29–32). Addition of BAFF multi-trimers into DNA vaccines encoding HIV envelope proteins strongly enhances the neutralizing activity of anti-HIV Abs in mice (33). This adjuvant activity is thought to result from the enhanced BAFF-mediated survival of Envelope-specific B-cells. In addition, BAFF might amplify response to TD antigens by regulating T_FH_ expansion as shown in mice (34) and recently suggested in healthy individuals (35). Through its production by follicular dendritic cells (FDC) and T_FH_ in GC, BAFF might thus exert a physiological role on T_FH_ during response to natural or vaccine TD antigens (36, 37). During the acute phase of HIV infection, a cytokine storm develops (38, 39) that could favor abnormal survival of low affinity B-cells as well as early differentiation of marginal zone (MZ) B-cells into plasma cells. Elevated levels of blood BAFF were found in pathogenic HIV/SIV infection (40–43). In primary HIV-infected individuals, intermediate and non-classical monocytes as well as conventional dendritic cells (DC) substantially contribute to high levels of blood BAFF (41, 42). In contrast, plasmacytoid DC (pDC) do not release BAFF upon exposure to TLR7/8 or TLR9 ligands, infectious rotavirus and either infectious or AT2-inactivated HIV-1 (41, 44). BAFF excess has been frequently associated with expansion/survival of self/poly-reactive B-cells during GC reaction (45–47) and more recently at the immature-transitional stage in mice (45). Therefore, BAFF overproduction during early HIV/SIV infection might be beneficial to B-cells producing broadly neutralizing Abs against HIV, a substantial fraction of them bearing self-/poly-reactive B-cell receptor (BCR) (48, 49). However, recent studies failed to demonstrate a beneficial effect of BAFF overexpression in their development. First, in a cohort of women followed pre- and post-infection by subtype C HIV-1, elevated plasma BAFF levels found at the hyper-acute phase of infection failed to predict the emergence of neutralizing Abs at 1 year post-infection (28). Second, in a cohort of vertically HIV-1 infected children, high levels of BAFF in progressors correlate with poor viral neutralizing activity (50).

The purpose of the present study has been to evaluate the effect of early BAFF blockade on SIV-induced changes in memory and GC B-cells, release of inflammatory cytokines, proportions and functions of T_FH_ and viral control. For this goal, we favored a soluble form of BAFF-R, BR3-Fc, as BAFF antagonist. This molecule binds to membrane and soluble BAFF in humans and in macaques but is devoid of antibody-mediated cell cytotoxicity. Thus, B-cell reduction in healthy macaques is slower and less severe with BR3-Fc than with anti-BAFF antibody, with protection of the memory B-cell compartment during the 1st month of treatment (51, 52). Our present results in SIV-infected macaques show that treatment with BR3-Fc affects the kinetics of inflammatory cytokine production and pDC renewal toward tissues. Despite similar proportions of plasma blasts in GC, lower levels of circulating SIV-specific Ig and anti-Tetanus Toxoid IgG were observed in Treated macaques, suggesting that BAFF blockade impairs the survival of plasma cells. This study also details expression of BAFF-R and TACI on various spleen B-cell subsets, in particular on atypical memory B-cells, during acute SIV infection and describes for the first time the consequences of BAFF blocking during the early steps of blood, spleen, and intestinal antiviral B-cell response in a macaque model of SIV infection.

## Materials and Methods

### Animals, Infection, and BR3-Fc Treatment

**Figure 1:**
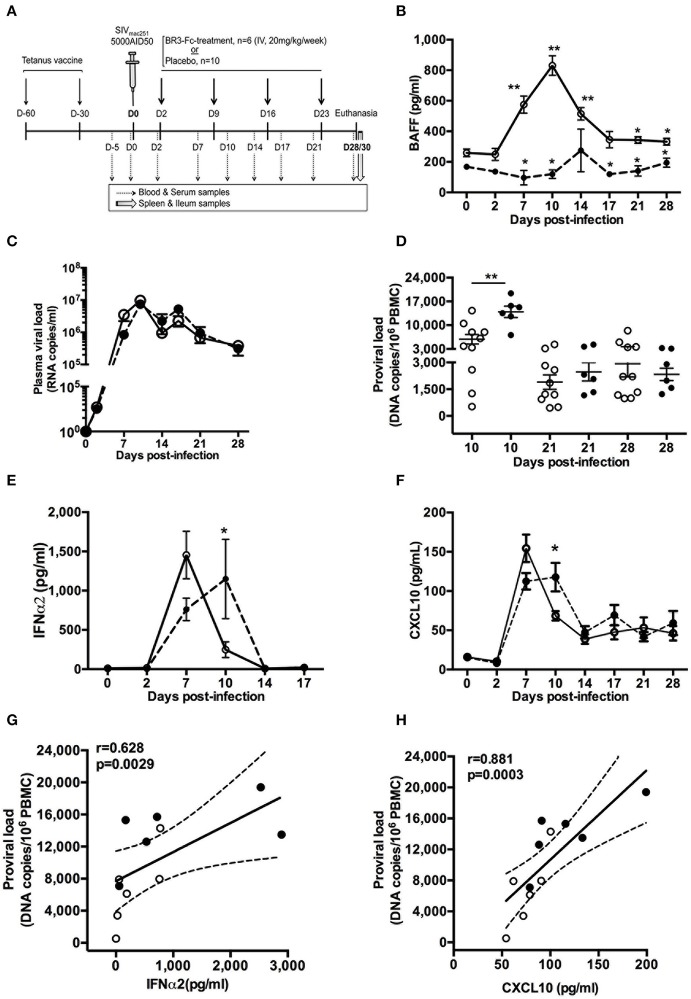
BR3-Fc treatment impairs kinetics of inflammatory cytokine production but not that of plasma viral load. **(A)** All macaques were vaccinated with Tetanus Toxoid (TT) vaccine 60 and 30 days before infection and then intravenously infected with 5000 AID50 SIV_mac251_. Six macaques were treated by soluble BR3-Fc (20 mg/kg/week) for 4 weeks (*BR3-Fc treated group*). Ten vehicle-treated or untreated SIV-infected macaques constituted the Placebo group. Blood and serum were collected before infection, every 3 dpi (dotted arrows) and at euthanasia. Spleen and terminal ileum were taken at necropsy from all macaques (gray arrow). **(B)** Plasma BAFF levels were quantified by ELISA prior to infection (baseline value, D0) and at different time points after infection. Symbols represent the Mean ± SEM value for Placebo (*open circle, plain line*) or Treated (*black circle; dotted line*) groups. For Placebo, statistical comparison between values at D0 and at every time point post-infection was carried using the Wilcoxon *t*-test *p < 0.05, **p < 0.001 (*asterisks above the plain line*). Statistical comparison between Placebo and Treated groups at each time point was carried out using the Mann Whitney non-parametric test *p < 0.05, **p < 0.001 (*asterisks above the dotted line*). **(C)** Plasma viral loads were measured at different time points for both groups. No statistical difference between groups was observed. **(D)** Pro-viral loads in PBMC were quantified at 10, 21, and 28 dpi. Statistics were carried out using the Mann Whitney non-parametric test, **p < 0.001. **(E,F)** ELISA was used to quantify plasma levels of IFNα2 **(E)** and CXCL10 **(F)** prior to infection and every 3 dpi for both groups. Statistical comparison between groups at every time point was carried out using the Mann Whitney non-parametric test, *p < 0.05. For **(B,C,E,F)** symbols represent the Mean (±SEM) value at each time point. For **(D)**, each dot represents one macaque of either Placebo (*open circle, plain line*) or Treated (*black circle; dotted line*) group. Bars represent Mean values ±SEM. **(G,H)** Correlations between the pro-viral load and levels of plasma IFNα2 **(G)** and CXCL10 **(H)** at 10 dpi are shown. Each dot on the graphs represents one macaque from Placebo (*open circle*) or Treated (*black circle*) group. Spearman rank test was used for statistical analysis. *rho* and *p*-values are given.

Sixteen adult male cynomolgus macaques (*Macaca fascicularis*), each weighing about 4 kg and aged about 34–45 months, were imported from Mauritius (SILABE, France) and housed in the accredited animal facilities of the Infectious Disease Models and Innovative Therapies (IDMIT) Infrastructure, Fontenay-aux-Roses, France. Non-human primates were handled in accordance with national regulations (Commisariat à l'Energie Atomique et aux Energies Alternatives (CEA) Permit Number A 92-32-02), in compliance with Standards for Human Care and Use of Laboratory of the Office for Laboratory Animal Welfare under Office for Laboratory Animal Welfare Assurance number A5826-01, and the European Directive (2010/63, recommendation N°9). The study was approved by the Ministère de l'Education Nationale, de l'Enseignement Supérieur et de la Recherche (France) and the Comité d'Ethique en Expérimentation Animale n°44 under the reference 2015121509045664 (APAFIS#3178). Animals were fed standard monkey chow diet supplemented daily with fruit and vegetables and water *ad-libitum*. Overall animal health was monitored daily by care staff and veterinary personnel. The MHC haplotype was determined as previously described (53), and animals negative for H6 haplotypes were distributed into two experimental groups in a balanced way (Table S1). All animals were immunized by intramuscular injection (0.5 mL per injection, pre-filled syringe) with the commercial Tetanus Toxoid (TT) vaccine from Sanofi Pasteur (Sanofi Pasteur SA, Lyon, France), 60 and 30 days before infection. All animals were then intravenously inoculated with 5000 AID50 of a SIVmac251 stock (54) for 28–30 days. The Treated group (*n* = 6, Treated) received 20 mg/kg of BR3-Fc molecule, an antagonist of soluble and membrane BAFF (Biogen Idec, USA) on days 2, 9, 16, and 23 post-infection (dpi) by slow intravenous infusion as recommended (52). Six Placebo macaques were concurrently treated by vehicle after infection. Four additional untreated SIV-infected macaques were then considered in the Placebo group. All animals were sacrificed between 28 and 30 dpi. The experimental protocol is detailed in Figure 1A. All animals were sedated with ketamine chlorhydrate (Merial SAS, Villeurbanne, France) before immunization, sample collection and necropsy. Samples from spleen and terminal ileum from healthy non-infected macaques previously enrolled in vaccine trials and necropsied at least 6 months after the last injection were used as controls for flow cytometry (FCM) and immunohistochemistry (IHC) experiments.

### Sample Collection and Processing

Before infection and at 2, 7, 10, 14, 17, 21, and 28/30 dpi, blood was collected into serum clot activator tubes or into K3-EDTA tubes (both from Greiner Bio-One, Frickenhausen, Germany) for plasma/serum sampling and complete blood count. Aliquots of serum and plasma samples were kept frozen at −80°C until use. PBMC were obtained from peripheral blood, collected into K3-EDTA tubes, before infection and at days 7, 10, 14, 21, and 28/30 pi and kept frozen in liquid nitrogen until use. Two dry pellets of PBMC (3 × 10^6^ cells/pellet) were prepared at every time point. Spleen, peripheral lymph nodes (axillary and inguinal) and terminal ileum were immediately collected at necropsy under the supervision of veterinarians. Spleen biopsies were formalin-fixed and paraffin-embedded as previously described (40, 55). Splenic and nodular mononuclear cells were obtained by mechanical disruption in complete medium (RMPI 1640-glutamax medium with 1 mM sodium pyruvate, 100 μg/ml streptomycin, 100 UI/ml penicillin, 10 mM HEPES buffer, 2 mM non-essential amino acids and 10% heat-inactivated FCS (all from Invitrogen, Life Technologies SAS, Saint Aubin, France), filtered through a 70 μm-pore-size cell strainer and further purified by Ficoll gradient centrifugation. After washes in complete medium, two dry pellets of 3 × 10^6^ cells were prepared for each tissue and kept at−80°C. The remaining cells were kept frozen in 90% FCS/10% DMSO in liquid nitrogen until use. Biopsied terminal ileum was cut into small pieces prior to incubation under agitation with 1 mM DTT and 20 mM HEPES in PBS 1X (two-times for 20 min at 20°C) and next with 20 mM EDTA and 20 mM HEPES in PBS 1X (two-times for 20 min at 20°C). After two washes with PBS 1X, biopsy fragments were submitted to two sequential incubations in complete medium with 0.5 mg/ml collagenase (from *Clostridium histolyticum*) and 0.1 mg/ml DNAse I (both from Sigma, France) for 30 min under permanent agitation at 37°C. After final tissue disruption, cell suspension was passed through a 70 μm-pore-size cell strainer and washed before preparing dry pellets (3 × 10^6^ cells) and frozen cells as described above for the spleen.

One inguinal lymph node was collected on 8 dpi from 8 macaques (4 Placebo and 4 BR3-Fc Treated) to determine pDC counts and follow mobilization (Ki67 staining). After having eliminated fat and connective tissues, cell suspensions were prepared by mechanical disruption in complete medium and immediately used for FCM.

### Hematology Analyses and Viral Load Determination

Blood cell formula and count as well as hemoglobin concentration and hematocrit were determined on K3-EDTA collected blood using a HMX A/L (Beckman Coulter, Villepinte, France). The SIV copy number in plasma was determined using quantitative real-time PCR, as previously described (56). For pro-viral load determinations in PBMC or tissues, total DNA was extracted from dry pellets. PCR was performed in duplicates with a limit of detection of 60 copies/ml for plasma viral load (pVL) (56) and 10 DNA copies per million cells for pro-viral loads (57).

### Quantification of Plasma Cytokine, Immunoglobulins, and Antigen-Specific Antibodies

Plasma BAFF, CXCL13, and CXCL10 were detected using Quantikine® ELISA kits (Bio-Techne, Lille, France). Plasma IFNα2 was detected using the Cynomolgus/Rhesus IFNα ELISA kit from PBL Assay Science (Bio-Techne). Prior to BAFF quantification, residual BR3-Fc molecules (free or bound to BAFF) were depleted from plasma using Pierce Protein A/G Plus agarose (Pierce, Life Technologies) according to the manufacturer's instructions. Anti-SIV Abs were detected in plasma using Genscreen HIV1/2 ELISA kit, version 2 (Bio-Rad Laboratories, Redmond, WA). Total IgM and IgG were quantified in plasma or culture supernatants by ELISA as previously described (55). Homemade ELISA was used to quantify plasma anti-TT IgG. In brief, plates were coated with 5 μg/ml Tetanus Toxoid (*Clostridium tetani*, Calbiochem, Merck Millipore), and 1 μg/ml goat anti-monkey IgG HRP conjugated Ab (Abd Serotec, Bio-rad) was used as detection antibody. For each macaque, plasmas collected before and at different dpi were tested simultaneously and run in duplicates. Results are expressed as mean OD value (SIV or TT-specific Abs) or as mean concentration (cytokines, total IgM/G).

### Flow Cytometry Analyses

Panels of Abs used for multi-parameter FCM are listed in Tables S2, S3. Optimized concentrations were predetermined for each Ab. For the detection of transcription factors (Bcl-6, Ki67), CD1c or surface receptors of BAFF (BAFF-R, TACI) and intracellular detection of IFNγ, relevant isotype controls used were indicated in gray. After rapid thawing at 37°C and two washes in complete medium, different staining procedures were used. For surface staining: 1 to 2 × 10^6^ cells in staining buffer (PBS 1X plus 0.5% BSA and 2 mM EDTA) were incubated with Live/Dead fixable blue stain (Invitrogen) for 30 min at 4°C before addition of 5% (vol/vol) heat-inactivated human AB serum for an extra 15 min at 4°C. After washing, cells were labeled with appropriate Abs diluted in staining buffer for 30 min at 4°C then washed and fixed with 0.5% paraformaldehyde. For the detection of transcription factors, cells were first surface stained as above, and then fixed and permeabilized with the eBiosciences™ FoxP3/Transcription Factor staining buffer set (Thermo Fischer Scientific) before intracellular staining with appropriate monoclonal Abs for 45 min at 4°C. Cells were washed twice and fixed in 0.5% paraformaldehyde. For the detection of intracellular cytokines, cells (2 × 10^6^/ml) were stimulated for 5 h at 37°C in 5% CO_2_ with PMA (50 ng/ml) and ionomycin (1 μg/ml) in the presence of Brefeldin A (BFA, 10 μg/ml) during the last 4 h before staining. After surface staining as above, cells were fixed with 2% PFA and treated with the BD cytofix/cytoperm kit before intracellular staining with anti-cytokine Abs. Events were acquired on a BD LSRII and data were analyzed using the Kaluza® Flow Analysis Software (v1.5, Beckman Coulter, Inc). Sphero™ Rainbow calibration particles (BD Biosciences) were used for daily calibration of the flow cytometer. Unstained cells and single-color beads were used for calculating the compensation matrix. Regarding PBMC, the absolute number of cells per blood microliter was calculated by multiplying the complete blood count of mononuclear cells (assessed independently on whole blood) by the percentage of cells among CD45^+^ events. The various leukocyte populations were identified according to Panel 1, Table S2.

### *In vitro* Production of Immunoglobulins by Purified Spleen B-Cells

B-cells were purified from spleen cell suspension using non-human primate CD20 Microbeads (Miltenyi Biotech, MACS, Paris, France) according to the manufacturer's instructions. Only B-cell fractions containing >95% CD20^+^ cells were used. Splenic B-cells (2 × 10^6^cells/ml) were cultured for 10 days at 37°C with complete medium, 20 ng/ml IL2 plus 50 ng/ml IL10 (both cytokines from Bio-Techne), or 50 ng/ml IL-21 (MACS), with or without 100 ng/ml CD40MegaLigand (CD40ML, Coger, Paris, France) and 10μg/ml CpG-B (ODN2006, InvivoGen, France). IgM and IgG concentrations were determined in cell-free culture supernatants by specific ELISA as previously described (55). Results are expressed as mean concentration (ng/ml) of duplicate values.

### Immunohistochemistry and Digital Image Analysis

Sections (4 μm-thick) were cut from formalin-fixed paraffin-embedded spleen blocks. Sections were subject to dewaxing, antigen retrieval, saturation and staining with various monoclonal and polyclonal antibodies (Table S4) on a Leica-Bond III/Max autostainer platform (Leica Biosystems Nanterre, France). Detection of primary Ab binding was performed with either bond compact polymer Refine detection (DAB, brown) or red Refine detection (Fat Red, red) kits. For double staining (Red/brown), Leica Chromoplex dual detection kit was used. All these kits contain substrate chromogen and hematoxylin counterstain.

Images of full section were generated on a Lamina Multilabel Slide Scanner (PerkinElmer, Gif s/Yvette, France), using the brightfield scan mode. Digital images were opened in Pannoramic Viewer software (v1.11.4, 3DHistech, Budapest, Hungary), and areas of interest were manually annotated using the drawing tools. For each section, all or at least 10 random areas were extracted from the main scan for quantitative analysis. The software automatically calculated the sizes of the whole tissue and of the various selected areas (in μm^2^). Quantification of positively labeled cells was performed with computer assisted image analysis using Inform (v2.3, 3DHistech) or manually with Photoshop CS6 (Adobe Systems Inc.) software by two independent investigators. Mean values of positive cells per area or per mm^2^ of tissue were calculated for each section and each macaque.

### Sorting of T_FH_ and Memory CD4^+^ T-Cell Subsets

According to the availability of total spleen cells and frequencies of T_FH_, sorting experiments have been performed on 4 Placebo and 4 Treated macaques. After thawing, total CD4^+^ cells were purified with CD4 magnetic beads (MACS) from 250 to 300 × 10^6^ spleen cells. CD4^+^ cells were then stained with the T_FH_ sorting panel (Panel 9, Table S3). Using a bio-contained ARIA III equipped with a 100 μm nozzle, naïve T-cells (CD45RA^+^CD3^+^CD4^+^) and three memory (CD45RA^lo^) CD4^+^ T-cell subsets (mCD4^+^) were sorted according to the intensity of PD1 and CXCR5 expression: T_FH_ (PD1^hi^CXCR5^hi^), CXCR5^int^ (PD1^int^CXCR5^int^), and CXCR5^lo^ (PD1^lo^ CXCR5^lo^). The purity of each sorted subset was higher than 95%.

### Cytokine Production, T-Cell Proliferation, and Survival

Sorted T-cell subsets were labeled with 2.5 μM Cell Trace CFSE cell proliferation kit (Invitrogen) for 10 min at 20°C and washed before culture. For each subset, 2 × 10^5^ cells were cultured with medium or CD2/CD3/CD28 stimulating beads (MACS, 1:1 ratio) in a final volume of 200 μl. On day 5, supernatants were harvested for cytokine detection, and proportions of live and proliferating CD4^+^ T-cells were determined after staining with Live/Dead Fixable Dead cell stain kit (Invitrogen) and CD4 antibody.

### B- and T-Cell Co-cultures

Total B-cells were sorted from spleen cell suspensions from the 8 SIV-infected macaques (autologous) and from two healthy non-infected macaques (heterologous). B-cells (5 × 10^4^/well) were cultured with 5 × 10^4^ cells of each sorted mCD4^+^ T-cell subset in 200 μl final medium in the presence of 250 ng/ml staphylococcal enterotoxin B (SEB, Sigma). Cultures of autologous or heterologous B-cells with medium or SEB alone were performed as controls. Each culture condition was run in duplicates or triplicates according to T-cell recovery after sorting. On day 7, supernatants were harvested and analyzed for total IgG by ELISA. Each culture condition was tested in duplicates and results are expressed as mean concentration. Results were further expressed in Fold Increase (FI), calculated as the ratio between IgG concentrations in culture of B-cells plus SEB, with and without CD4^+^ T-cells.

### ELISA and Multiplex Cytokine Assay

Monkey IL21 and IFNγ were detected in 5 days supernatants from T-cell cultures using ELISA kits (Mabtech AB, Nacka Strand, Sweden). CXCL13, IL2, IL4, IL6, IL17A, IL10, and BAFF were measured in T-cell supernatants using a magnetic Luminex Kit (Bio-Techne). Data were acquired using a Bioplex-200 and analyzed with the Bioplex Manager Software (Bio-rad). Results are expressed as mean concentration (pg/ml) of duplicates.

### Statistical Analysis

All data were graphed and analyzed using Graphpad Prism (GraphPad Software, San Diego, CA). Bar graphs represent mean and SEM, whereas boxes in plots show the median values and 5th and 95th percentiles. For pairwise comparisons, data were analyzed using Mann-Whitney *U*-test (unpaired, 2-tailed unless otherwise indicated) or Wilcoxon matched pairs test. Two-way ANOVA was used for multiple comparisons. Correlation coefficients were calculated using the Spearman rank test. Statistical significance is denoted on each figure by asterisk as ^*^p < 0.05; ^**^p < 0.01, ^***^p < 0.001 and ^****^p < 0.0001.

## Results

### BAFF Blockade Delayed the Peak of SIV-Induced Circulating Inflammatory Cytokines

To assess the impact of BAFF blockade during acute SIV infection, we compared 2 groups of SIV-infected macaques, a first group of 6 SIV-infected macaques (Treated group) receiving, every week for 4 weeks, the BR3-Fc molecule according to the experimental protocol depicted in Figure 1A. The second group, hereafter referred to as the Placebo group, comprised 10 macaques that were treated by vehicle (*n* = 6) or left untreated post-infection (*n* = 4).

Consistent with our previous data (40), plasma BAFF level increased after 2 dpi in the Placebo group and peaked at 10 dpi with a 3.3-fold increase compared to baseline values (942 ± 60 vs. 284 ± 37 pg/ml) (Figure 1B). From 17 to 28 dpi, the average BAFF level remained significantly higher than the baseline value. As expected, concentrations of free plasma BAFF were in the range of baseline values in BR3-Fc treated animals at any time. Except at 14 dpi, BAFF levels significantly differed between Placebo and Treated groups. However, BAFF blockade did not change the kinetics of plasma viral load (pVL) (Figure 1C), which peaked at 10 dpi in both groups (7.46 ± 1.93 × 10^6^ and 9.71 ± 1.67 × 10^6^ RNA copies/ml in Placebo and Treated macaques, respectively). In contrast, the pro-viral load at 10 dpi was significantly higher in the Treated than in the Placebo group (2.4-fold, 13,932 ± 1,670 vs. 5,836 ± 1,382 copies/10^6^ PBMC) but similar afterwards (Figure 1D). Similar average counts of CD4^+^ T-cells (321 ± 76 cells/μl and 306 ± 41 cells/μl, respectively) and proportions of activated CD4^+^ T-cells (3.4 ± 1.2% and 3 ± 0.8% Ki67^+^ cells, respectively) were observed in PBMC of Placebo and Treated groups at 10 dpi (Figure S1). Due to the key role of inflammation in disease progression, levels of plasma IFNα2 and CXCL10 were also compared between the two groups. In the Placebo group, IFNα2 level peaked at 7 dpi (1,454 ± 302 pg/ml) and rapidly decreased at 10 dpi (223 ± 94 pg/ml) (Figure 1E). The kinetics was slightly different in the Treated group where the peak was reached at 10 dpi only (1,149 ± 505 pg/ml). For both groups, levels returned to baseline values at 14 dpi. Similar results were observed for CXCL10 level, which peaked at 7 dpi in the Placebo group and was still significantly higher at 10 dpi than before infection (76.3 ± 7.1 and 16 ± 2.4 pg/ml, respectively) (Figure 1F). Compared to Placebo, plasma CXCL10 in the Treated group was reduced by 23% at 7 dpi (112.4 ± 10.4 vs. 146.6 ± 23.2 pg/ml) but significantly increased by 54% at 10 dpi (117.8 ± 18.2 vs. 76.4 ± 3 pg/ml) (Figure 1F). Pro-viral load in PBMC at 10 dpi correlated with levels of IFNα2 (Figure 1G) and CXCL10 (Figure 1H).

### BAFF Blockade Alters pDC Turnover

**Figure 2:**
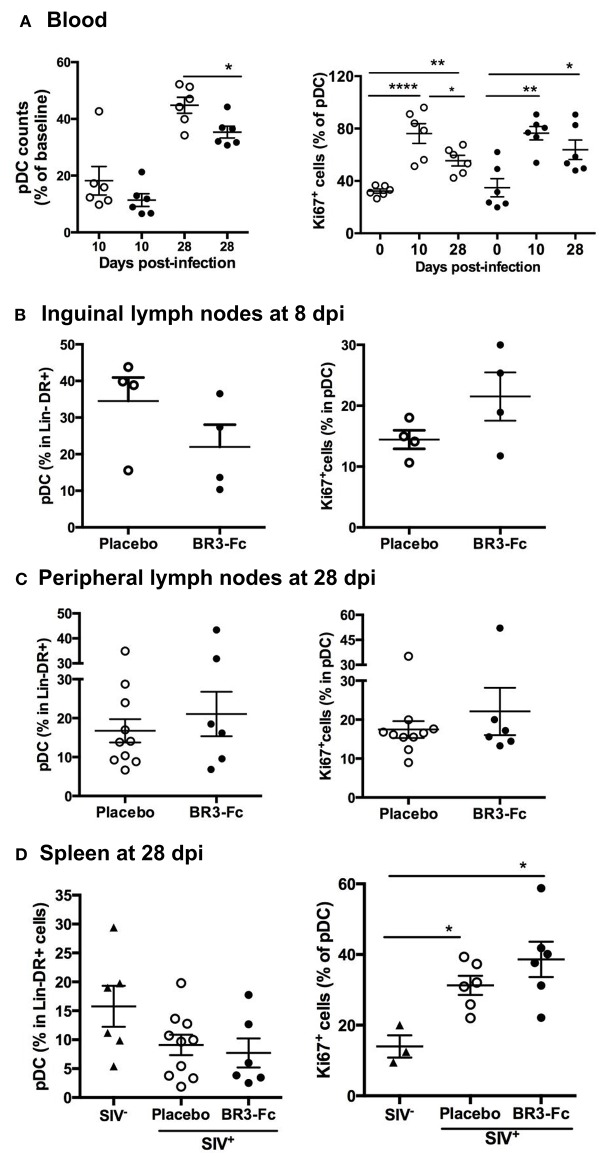
Blockade of BAFF changes early pDC renewal in tissue. (**A**, left panel) Change in blood pDC counts in Lin^−^DR^+^ lymphocytes, expressed as the percentage of baseline value, was calculated as the ratio between the numbers of pDC at 10 or 28 dpi and that prior to infection (D0) for each animal. Statistics were carried out using the Mann Whitney non-parametric test, *p < 0.05. (**A**, right panel) The percentage of pDC expressing Ki67 was determined prior to infection (D0), at 10 dpi (D10) and at 28 dpi (D28). For each group, statistics were carried out using the Wilcoxon matched-pairs signed rank test, *p < 0.05, **p < 0.01, ****p < 0.0001. **(B)** Percentages of pDC in Lin^−^DR^+^ cells (left panel) and of Ki67^+^cells in pDC (right panel) were determined in cell suspensions from inguinal lymph nodes collected at 8 dpi from 4 Placebo and 4 Treated macaques. Similar quantifications were performed in cell suspensions from peripheral lymph nodes **(C)** or spleen **(D)** collected at 28 dpi from Placebo and Treated groups. Each dot represents one macaque of either Placebo (*open circle*) or Treated (*black circle*) group. Bars represent Mean values ± SEM. Statistics were carried out using the Mann Whitney non-parametric test, *p < 0.05.

Plasmacytoid DC being key actors of SIV/HIV-induced inflammation at the acute phase, we evaluated the numbers of circulating pDC in both groups of macaques (Table S2, Panels 2 and 3). Compared to baseline, the average counts of circulating pDC in Treated and Placebo groups decreased by 89 and 82% at 10 dpi, respectively (Figure 2A, left panel). At 28 dpi, counts of pDC were significantly lower in Treated compared to Placebo group, and represented 35 and 45% of baseline values, respectively (Figure 2A). However, similar percentages of Ki67^+^ cells in pDC were present in both groups at 10 or 28 dpi (Figure 2A, right panel), indicating comparable pDC mobilization into blood (58–60). Considering that BAFF deprivation might alter pDC recruitment into lymphoid organs, we next compared the proportions of pDC in Lin^−^DR^+^ nodular cells from 4 macaques of each group at 8 dpi (Figure 2B, left panel). Compared to Placebo, we found a trend toward decreased pDC proportions (21.9 ± 6.1 vs. 34.5 ± 6.4%) but more Ki67^+^ pDC in Treated macaques (21.5 ± 4 vs. 14.4 ± 1.5%) (Figure 2B, right panel). However, these differences did not reach statistical significance and were no longer observed at 28 dpi (Figure 2C). Compared to uninfected macaques, the average proportions of spleen pDC in Lin^−^DR^+^ cells at 28 dpi tended to be lower in both groups of SIV-infected macaques with significantly more Ki67^+^ pDC being present (Figure 2D, right panels). Compared to Placebo, a trend toward higher proportions of Ki67^+^pDC was observed in spleen of Treated macaques. Thus, we infer that blocking BAFF enhances SIV-induced pDC loss in lymphoid organs that is partially compensated by sustained recruitment.

### BAFF Blockade Changes the Composition of the Circulating B-Cell Pool

**Figure 3:**
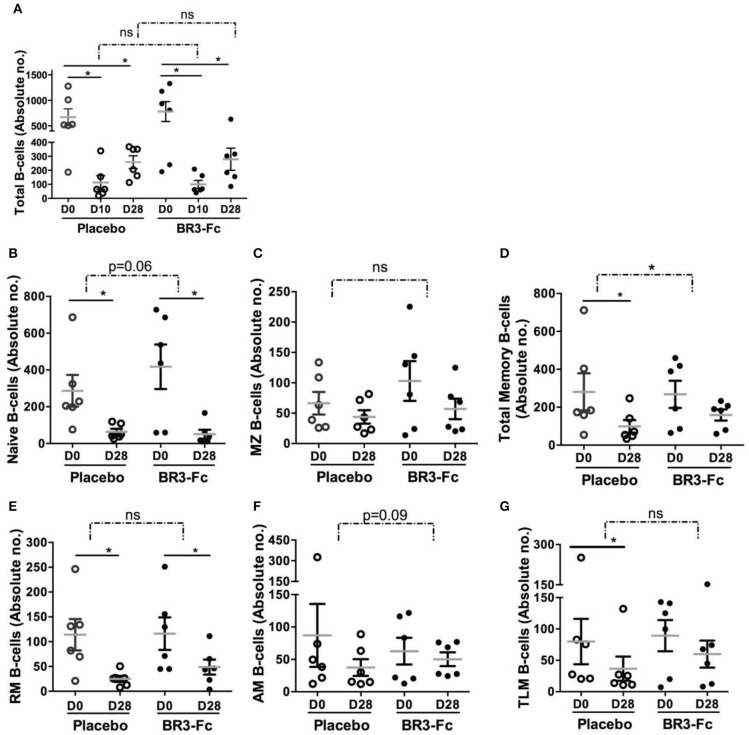
BR3-Fc treatment reduces SIV-induced loss in blood memory B-cells. Blood B-cell phenotype was determined by Flow cytometry and absolute numbers of every B-cell subset were calculated according to the whole blood cell count. Samples from 6 Placebos and 6 Treated macaques were compared. **(A)** Absolute numbers of total B-cells were plotted for each macaque before infection (D0) at 10 dpi (D10) and 28 dpi (D28). For **(B–G)**, only absolute numbers at D0 and D28 were plotted. Each dot on the graphs represents one macaque of Placebo (*open circle*) or Treated (*black circle*) group. Bars represent Mean values ± SEM. Statistics were first carried out to compare values at 10 (D10) and 28 (D28) dpi with those prior to infection (D0) for each macaque using the Wilcoxon matched-pairs signed rank test. Significant statistical values are indicted (*plain line*, *p < 0.05). B-cell loss corresponding to difference between absolute numbers at D10 and D0 (A) and D28 and D0 (all panels) was also compared between groups using the Mann Whitney non-parametric test. Statistical values are indicated for each panel (*dotted line and p-value*, *p < 0.05). *ns*: not significant.

Using polychromatic staining (Table S2, Panel 4) and the gating strategy depicted in Figure S2, we identified various circulating B-cell subsets. SIV infection induced a substantial leukopenia at the peak of viral load due to rapid relocation of leukocytes at sites of virus replication and inflammation. In particular, we have previously established that decrease in total B-cells (CD19^+^CD20^+^) at the peak of viral load is followed by a progressive, but frequently incomplete, reconstitution at 1 month post-infection (55). Thus, we compared changes in absolute numbers of total B-cells and various B-cell subsets in both groups of macaques. Treatment by BR3-Fc did not modify the SIV-induced decrease in total B-cells at 10 dpi (87 vs. 93% in Placebo) or at 28 dpi (64 vs. 62% in Placebo) (Figure 3A). Consistent with BAFF being mandatory for optimal B-cell differentiation into mature B-cells (61, 62), numbers of naïve (sIgD^+^CD21^+^CD27^−^) and MZ (sIgD^+^CD21^+^CD27^+^) B-cells were further decreased in Treated macaques (Figures 3B,C). Meanwhile, total memory B-cells were significantly more numerous in Treated macaques compared to Placebo (41 vs. 65% of baseline values) at 28 dpi (Figure 3D). Consistently, loss in RM B-cells (sIgD^−^CD21^+^CD27^+^) (Figure 3E), AM B-cells (sIgD^−^CD21^−^CD27^+^) (Figure 3F) and TLM B-cells (sIgD^−^CD21^−^CD27^−^) (Figure 3G) was reduced by 20, 27, and 10% in Treated group compared to Placebo group. However, these changes did not reach statistical significance.

### Impact of BAFF Blockade on Spleen and Terminal Ileum B-Cells

**Figure 4:**
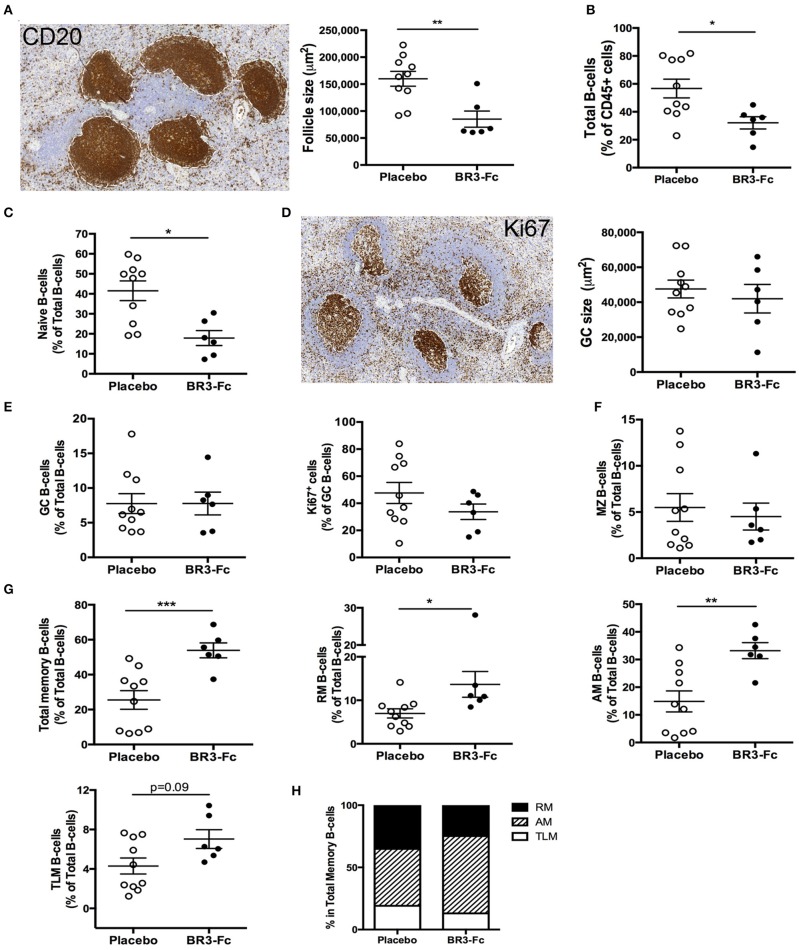
Higher proportions of activated memory B-cells in spleen and changes in BAFF receptor expression after BR3-Fc treatment. **(A)** Representative CD20 staining (left) on sections of spleen from one Placebo macaque. For each section, the size of all follicles was determined using Pannoramic Viewer software. Average size of follicles for each macaque was shown (right panel). **(B)** Proportions of total spleen B-cells (CD19^+^CD20^+^) in CD45^+^ cells were determined by multi-parameter FCM for each macaque. **(C)** Proportions of naïve B-cells among total B-cells were determined by multi-parameter FCM for each macaque according to the gating strategy shown in Figure S3. **(D)** Representative Ki67 staining (left) on sections of spleen from one Placebo. The average size of GC was determined using Pannoramic Viewer software for each macaque (right panel). **(E)** Proportions of GC B-cells in total B-cells (left panel) and of Ki67^+^ cells among GC B-cells (right panel) in spleen cell suspensions. **(F)** Proportions of MZ B-cells in total B-cells from spleen cell suspensions; **(G)** proportions of total, RM, AM, TLM memory B-cells in total B-cells from spleen cell suspensions; **(H)** graphs represent the relative frequencies of RM (*black*), AM (*hatched*) and TLM (*white*) B-cell subsets among total memory B-cells for each group of macaques. For **(A–G)**, each dot represents one macaque of Placebo (*open circle*) or Treated (*black circle*) group. Bars represent Mean ± SEM. Statistical comparison between groups was performed using Mann Whitney non-parametric test. Significant statistical values are indicated: *p < 0.05, **p < 0.01, and ***p < 0.001.

As viral replication, inflammation and adaptive antiviral B-cell response mainly occur in lymphoid organs, we next compared the B-cell compartment at 28 dpi in spleen from Placebo or Treated macaques. CD20 staining of spleen sections (Figure 4A) showed that the average number of follicles was similar in the two groups of animals (1.29 ± 0.2 and 1.37 ± 0.3 follicles/mm^2^, *not shown*). However, their average size was significantly reduced in Treated animals (1.9-fold, 85,163 vs. 159,830 μm^2^ in Placebo; Figure 4A), likely because follicular mantle zones were dwindled in BR3-Fc Treated macaques compared to Placebo. Consistently, lower proportions of total B-cells (CD19^+^CD20^+^) were found among total CD45^+^ cells in Treated macaques compared to Placebo (Figure 4B). Using polychromatic staining (Table S2, Panel 5) and the gating strategy depicted in Figure S3, we identified various spleen B-cell subsets. Consistent with thinner follicular mantle zones, proportions of naïve B-cells were significantly lower in Treated than in Placebo macaques (Figure 4C) Staining of spleen sections with Ki67 mAb showed that 84.2 ± 2.5 and 75.8 ± 5.3% of follicles contained active GC in Placebo and Treated animals, respectively, with GC having comparable average size (47,549 and 41,999 μm^2^) (Figure 4D). By FCM, we identified GC B-cells by their expression of BCL6 and showed that these cells were SIgD^−^ and expressed intermediate levels of CD21 and CD27 (Figure S3). Proportions of GC B-cells among total B-cells were similar in both groups (7.8 ± 1.6 vs. 7.8 ± 1.4%; Figure 4E). But a trend toward lower proportions of proliferating B-cells (Ki67^+^) was observed in Treated macaques compared to Placebo (33.7 ± 5.7 vs. 47.6 ± 7.7%, 29% of decrease). No difference in the intensity of BCL6 or Ki67 expression (geometric MFI) was concurrently observed (*data not shown*). Within the BCL6^−^ B-cells, MZ B-cells were CD21^hi^ but express intermediate levels of sIgD and CD27 (CD21^hi^CD27^int^sIgD^int^), and their average proportions did not vary between the two groups (5.5 ± 1.5 and 4.5 ± 1.5% in Placebo and Treated macaques, respectively) (Figure 4F). However, these proportions in SIV-infected macaques were significantly reduced compared to those in non-infected macaques (25.7 ± 5.3%, *n* = 6, *p* < 0.001 for Placebo and *p* < 0.004 for Treated macaques; Table S5). Meanwhile, average proportions of total memory B-cells were 2-fold higher in Treated macaques than in Placebo (53.9 ± 4.3 vs. 25.7 ± 5.2%, respectively) (Figure 4G). Among total memory B-cells, this change particularly benefits AM B-cells (Figure 4H). In terminal ileum of uninfected macaques, RM B-cells predominate over naïve and MZ B-cells (Table S6). SIV infection (Placebo group) was associated with reduced proportions of RM B-cells but increase in those of naïve and GC B-cells. These changes were not observed in Treated macaques while average proportions of proliferating (Ki67^+^) GC B-cells were 28% lower in Treated than Placebo macaques. Thus, BAFF blockade tends to dampen SIV-induced GC proliferation in spleen and terminal ileum.

### BAFF Blockade Differently Modulates BAFF-R and TACI Expression in Various Spleen B-Cell Subsets

**Figure 5:**
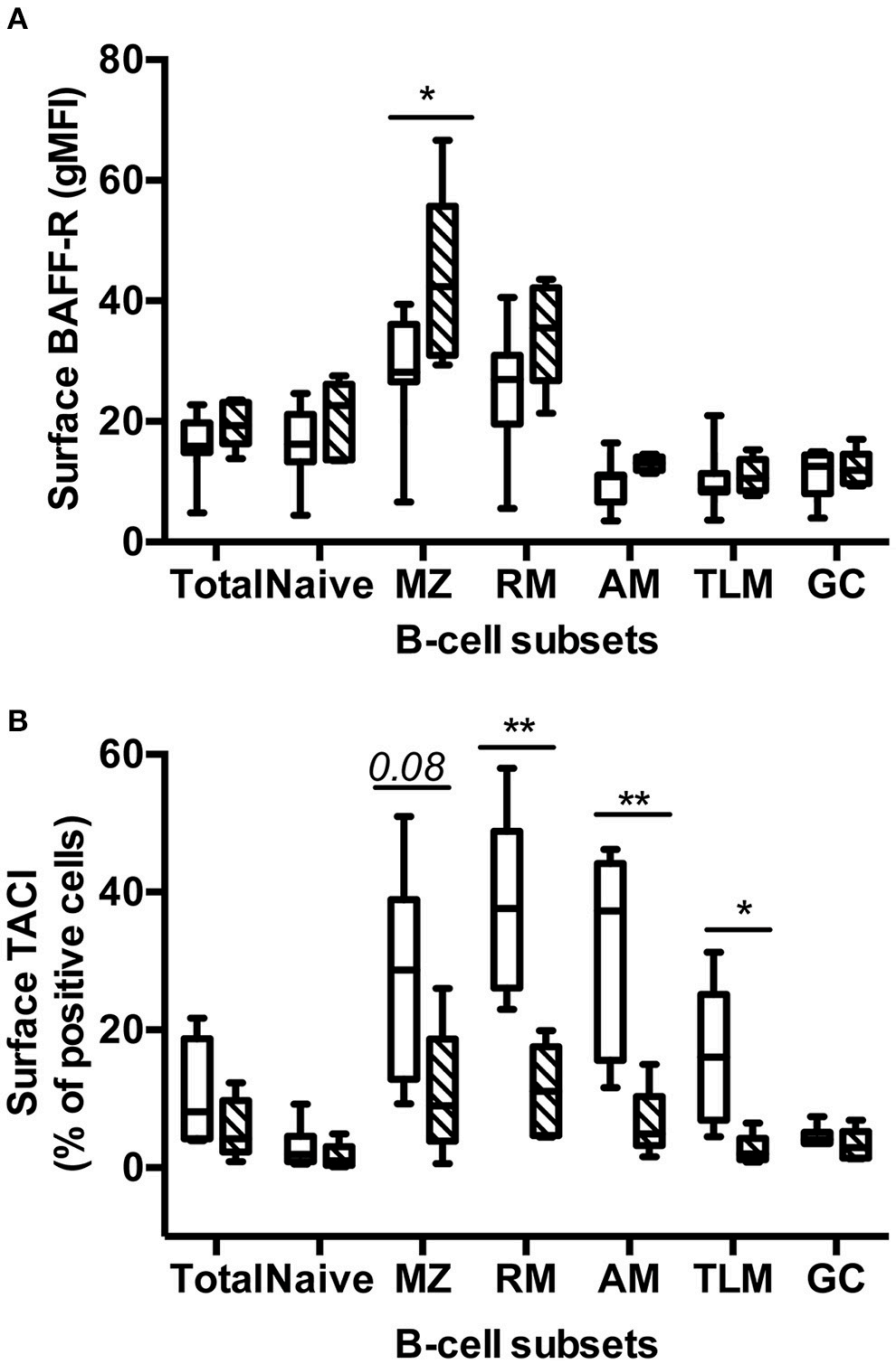
Treatment by BR3-Fc modifies the expression of BAFF-R and TACI by spleen B-cells. Surface expression of BAFF-R (**A**, gMFI) and TACI (**B**, % of positive cells) were analyzed in every B-cell subset from 7 Placebos (*white bar)* and 5 Treated macaques (*hatched bar)*. Results are presented in box-and-whiskers (5–95 percentile) plots with horizontal bars indicating median values. Statistical comparison between groups was performed using Mann Whitney non-parametric test. Significant statistical values are indicated: *p < 0.05 and **p < 0.01.

We next compared the surface expression of BAFF-R and TACI in the various spleen B-cell subsets (Table S3, panels 6 and 7). More than 90% B-cells in total and every spleen subset were BAFF-R positive in Placebo and Treated macaques (*data not shown*). However, the intensity of BAFF-R expression varied among subsets in Placebo macaques, the lowest intensities (gMFI) being observed in naïve, GC, AM, and TLM B-cells and the highest in MZ and RM B-cells (Figure 5A). A similar pattern of BAFF-R expression was observed in BR3-Fc treated macaques with the exception of BAFF-R expression being significantly increased in MZ B-cells (1.57-fold) and tending to be higher in RM B-cells (1.4-fold) compared to Placebo. Consistent with data in humans, TACI positive cells were more frequent in RM (38%) and MZ B-cells (26%) compared to naïve B-cells (3%) (Figure 5B). Moreover, we showed for the first time that 32% of AM and 16.5% of TLM B-cells were TACI^+^ in Placebo macaques, respectively. Treatment by BR3-Fc decreased TACI expression in all subsets, but this decrease was significant only in RM, AM, and TLM (71, 80, and 84% of decrease, respectively). Irrespective of BAFF blockade, <5% GC B-cells expressed TACI at their surface. Thus, most GC B-cells express low levels of BAFF-R and TACI in these SIV-infected macaques.

### Circulating Antibodies and Functional Response of B-Cells

**Figure 6:**
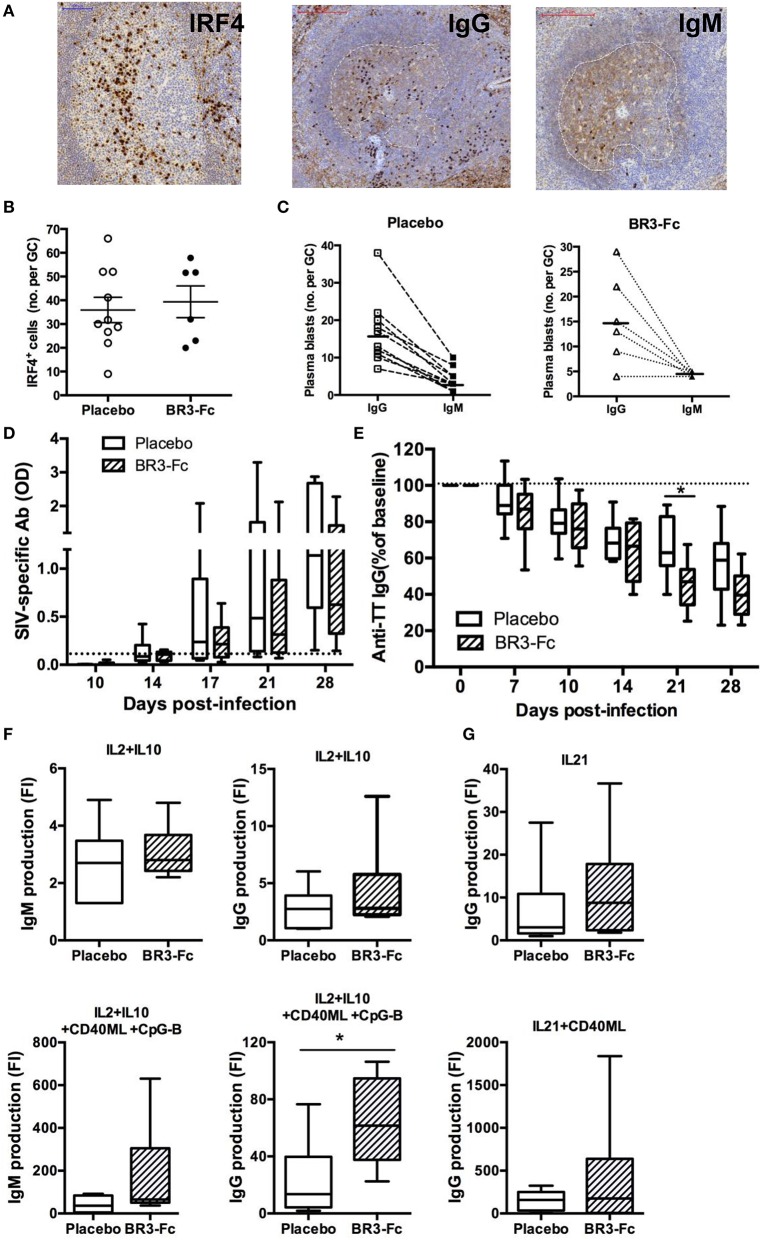
Higher production of IgG/M by spleen B-cells from Treated macaques than Placebo. **(A)** Representative IRF4, IgG and IgM staining on sections of one Placebo macaque. For IgG and IgM staining, a dotted white line surrounds the GC area. **(B)** Graph represents the mean number of IRF4^+^ cells per GC for each macaque as quantified using InFORM software. Each dot represents one macaque of Placebo (*open circle*) or Treated (*black circle*) group. Bars represent mean ± SEM. **(C)** For each macaque in Placebo (*n* = 10) or BR3-Fc Treated (*n* = 6) groups, mean number of IgG^+^ (*open square or triangle*) and IgM^+^ (*black square or triangle*) plasma blasts per GC was quantified using InFORM software. Bars represent mean value. Statistical comparison between groups was performed using the Mann Whitney non-parametric test (*p* = *ns*). ELISA was used to quantify anti-SIV antibodies **(D)** and anti-TT IgG **(E)** in plasma of Placebo (*white bars*) and Treated (*hatched bars*) macaques. Change in anti-TT IgG concentration was expressed as the percentage of baseline value (D0) for each animal. A dotted line indicates the cut off **(D)** or the 100% **(E)** value. Box-and-whiskers (5–95 percentile) plots with horizontal bars indicating median values are shown for each group. Statistical comparison between groups was performed using the Mann Whitney non-parametric test. Significant statistical values are indicated *p < 0.05. **(F,G)** B-cells purified from spleen cell suspensions of 10 Placebo (*open bars*) and 6 Treated (*hatched bars*) macaques were cultured for 7 days in the presence of medium, IL2+IL10 **(F)** or IL21 **(G)** with or without CD40ML or CD40ML and CpG-B. ELISA was used to quantify the concentrations of IgG and IgM (ng/ml) in cell-free supernatants. Results are expressed in fold increase (FI) that was calculated as the ratio between concentrations in the presence of each stimulus and medium. Box-and-whiskers (5–95 percentile) plots with horizontal bars indicating median values are shown. Statistical comparison between groups was performed using the Mann Whitney non-parametric test. Significant statistical values are indicated *p < 0.05.

We also evaluated the frequency of plasma blasts/cells in spleen by IHC using IRF4 and IgG/M staining as previously described (40, 55) (Figure 6A). We found similar frequency of IRF4^+^ plasma blasts per GC in Placebo and Treated macaques (36 ± 5.3 cells vs. 39 ± 6.7 cells, respectively) (Figure 6B). Moreover, GC plasma blasts more frequently expressed IgG than IgM (Figure 6C), with IgG/IgM ratios of 5.6 ± 1.2 and 4.8 ± 1.7 in Placebo and Treated macaques, respectively.

In agreement with our previous observation (55), anti-SIV antibodies were detectable in plasma from 17 dpi in SIV-infected macaques but their levels increased more in Placebo than in Treated animals. Compared to Placebo these levels were 35 and 45% lower in Treated macaques on 21 and 28 dpi, respectively (Figure 6D) but these differences did not reach significance. Levels of plasma anti-TT IgG were concurrently decreased by 31 and 28% at 21 and 28 dpi in Treated macaques compared to Placebo, but only the difference at 21 dpi reached significance (Figure 6E). In contrast, proportions of plasma blasts were comparable in both groups. We thus infer that reduced amounts of BAFF impair the survival of TT and SIV-specific plasma cells. According to our previous data showing that human BAFF preferentially potentiates CXCL13-mediated chemotaxis of memory B-cells (63), SIV-specific memory B-cells might also have limited capacity to reenter into GC in the absence of BAFF.

With the aim to compare intrinsic functional capacities of spleen B-cells from the two groups of SIV-infected animals, isolated B-cells were cultured in the presence of cytokines with or without CD40ML and/or CpG-B for 7 days before measuring Ig production in cell culture supernatants. Culture with IL2 plus IL10 similarly enhanced IgM concentrations in both groups of SIV-infected macaques (Figure 6F), whereas the addition of CD40ML/CpG to IL2+IL10 increased IgM and IgG levels by 1.7-fold and 2.7-fold in Treated macaques compared to Placebo. Similarly, IgG production in the presence of IL21 and IL21 plus CD40ML was 1.7-fold and 2.7-fold higher in Treated macaques than Placebo (Figure 6G). Enhanced IgG/M production in response to CD40ML in Treated animals is consistent with higher proportions of memory B-cells in spleen, with IL21 being a more potent IgG inducer than IL2 plus IL10 (64).

### Plasma Levels of CXCL13 and _TFH_ in Spleen

**Figure 7:**
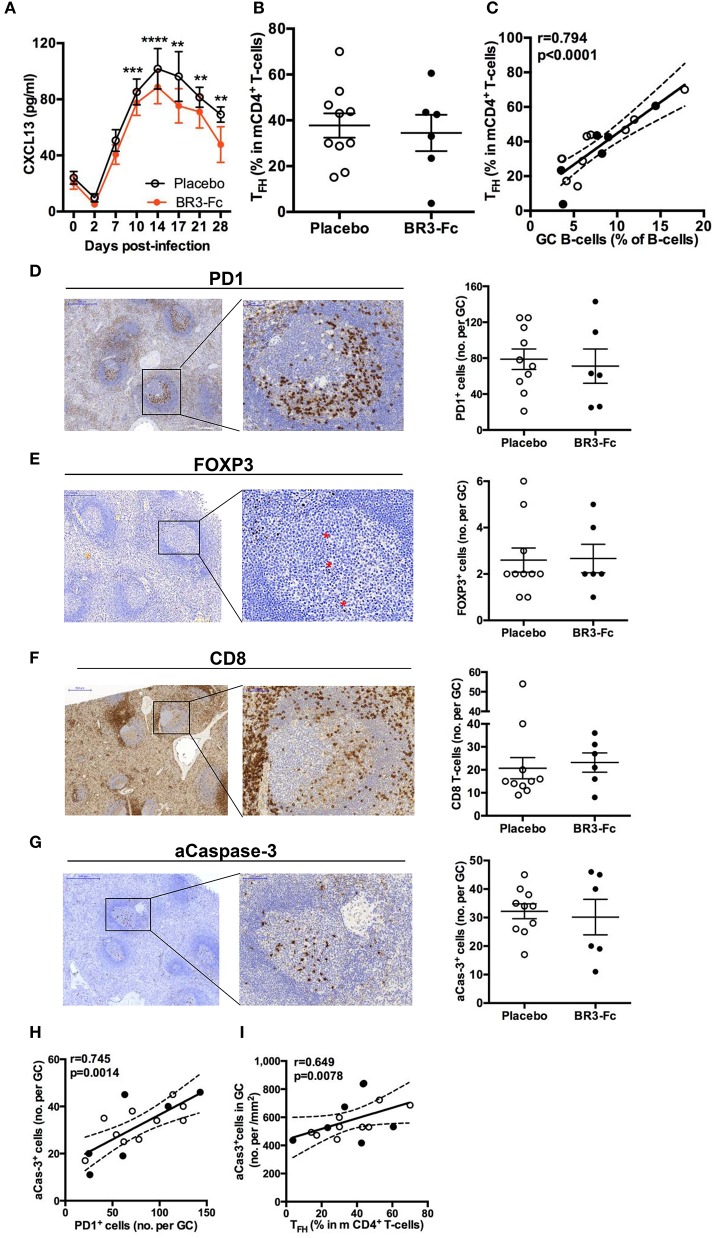
CXCL13 and T_FH_ in spleen of SIV-infected macaques. **(A)** Serum CXCL13 levels were quantified by ELISA prior to infection (baseline value, D0) and at different time points after infection. Symbols represent the mean value (±SEM) for Placebo (*open circle, plain line*) or Treated (*orange circle and line*) group. For every group, statistical comparison between values at D0 and at every time point post-infection was carried using the Dunn's multiple comparison test **p < 0.01, ***p < 0.001 and ****p < 0.0001. Statistical comparison between Placebo and Treated groups at each time point was carried out using the Mann Whitney non-parametric test (*p* = *ns* for all, *not shown*). **(B)** Proportions of T_FH_ in memory CD4^+^ T-cells (mCD4 T-cells) from spleen cell suspensions were determined by FCM. Each dot represents one macaque of Placebo (*open circle*) or Treated (*black circle*) group. Bars represent Mean ± SEM. Statistical comparison between groups was performed using the Mann Whitney non-parametric test (*p* = *ns*). **(C)** Correlations between the percentages of spleen GC B-cells and T_FH_ for all SIV-infected macaques are shown. Each dot represents one macaque of Placebo (*open circle*) or Treated (*black circle*) group. Spearman rank test was used for statistical analyses. *rho* and *p*-values are given. **(D–G)** Representative staining with PD1 **(D)**, FoxP3 **(E)**, CD8 **(F)**, and active Caspase-3 (aCas-3) **(G)** antibodies on sections from Placebo macaques are shown on the left part. For each marker, positive cells per GC were count by computer-assisted analysis, all GC being considered for every section. Each dot represents the mean percentage per GC for one Placebo (*open circle*) or Treated (*black circle*) macaque (*right part of panels*). Bars represent Mean values (± SEM). Statistical comparison between groups was performed using the Mann Whitney non-parametric test (*p* = *ns* for all). **(H)** Correlation between the numbers of aCas3^+^ cells and PD1^+^ cells per GC is shown. **(I)** Correlation between the number of aCas3^+^ cells per mm^2^ of GC and the percentage of T_FH_ in mCD4^+^ T-cells is shown. **(H,I)** Each dot represents one macaque of Placebo (*open circle*) or Treated (*black circle*) group. Spearman rank test was used for statistical analyses. *rho* and *p*-values are given.

CXCL13 is thought to be a reliable marker of GC reaction (27) and elevated levels of plasma CXCL13 have been reported in HIV-infected adults and children (25, 28, 65, 66). We thus compared serum CXCL13 levels in the two groups of SIV-infected macaques. In the Placebo group, CXCL13 level increased after 2 dpi and reached a plateau at 14 dpi before slowly decreasing thereafter (Figure 7A). Levels of CXCL13 thus peaked later than those of IFNα2, CXCL10 (7 dpi) and even BAFF (10 dpi) in the Placebo group (Figure 1). Whereas, CXCL13 level similarly increased until 10 dpi in Treated macaques, it more rapidly waned thereafter. The peak level was 15% lower at 14 dpi (88.7 ± 12 vs. 101.7 ± 14.4 pg/ml) in the Treated group than in the Placebo group. At 28 dpi, a 27% reduction (47.8 ± 12.7 vs. 65.3 ± 6.8 pg/ml) was observed. Neither of these differences reached statistical significance. Therefore, a faster contraction of GC might occur after 2 weeks of SIV infection when BAFF amounts are limited.

Production of highly specific B-cell clones is dependent on proper interactions between GC B-cells and T_FH_. Despite a strong decrease in splenic CD4^+^ T-cells in Placebo and Treated groups compared to uninfected controls (Figure S1C), the proportions of memory CD4^+^ T-cells (mCD4, CD45RA^−^) were not statistically different between the 2 groups of SIV-infected animals (71.3 ± 2.3% in the Placebo group and 71.4 ± 1.7% in the Treated group). Based on the combined expression of PD1, ICOS and BCL6 (Table S3, panel 8), T_FH_ were first characterized as PD1^hi^ICOS^hi^ mCD4 T-cells. Two other subsets of mCD4 T-cells expressed PD1 and ICOS at intermediate or low levels, respectively (Figure S4A). Accordingly, these three subsets were referred hereafter as to T_FH_, PD1^int^ and PD1^lo^. According to these criteria, we found 37.7 ± 5.3 and 34.5 ± 7.9% T_FH_ within mCD4 T-cells in Placebo and Treated macaques, respectively (Figure 7B). Irrespective of treatment, most T_FH_ expressed BCL6 (78 ± 5 and 89 ± 1.6% in Placebo and Treated groups, respectively) whereas only a proportion of PD1^int^ICOS^int^ were BCL6^+^ (21.6 ± 3.0 and 21.4 ± 2.6% in Placebo and Treated groups, respectively). Proportions of T_FH_ correlated with those of GC B-cells when all SIV-infected animals were considered (Figure 7C). To directly quantify T_FH_ within GC, we additionally used an IHC approach on spleen sections from all macaques using PD1 staining. In GC, T_FH_ preferentially accumulated in the light zone (Figure 7D). Quantification of these PD1^+^ cells was performed in all GC from one or two sections per animal, and an average number of PD1^+^ cells/GC was calculated and plotted in Figure 7D (right panel). When all animals were considered, the average number of PD1^+^ cells per GC did not differ between the two groups (78.8 ± 11.3 vs. 71.2 ± 19.1 cells/GC). Similar results were obtained when the number of PD1^+^ cells per mm^2^ of GC was considered (1,555 ± 142 vs. 1,730 ± 131 cells/mm^2^) (*data not shown*).

Expression of FoxP3, a reliable marker of regulatory (Treg) and follicular regulatory (T_FR_) T-cells, was also studied on spleen sections (Figure 7E). In contrast to PD1^+^ cells, FoxP3^+^ cells were essentially present in T-cell areas in all animals and rare in GC. Accordingly, the mean numbers of FoxP3^+^ cells per GC were 2.6 ± 0.52 and 2.7 ± 0.61 in Placebo and Treated groups, respectively (Figure 7E). A similar result was observed when mean numbers of FoxP3^+^ cells per mm^2^ of GC were considered (*not shown*). These data are in favor PD1^+^ cells in GC being T_FH_ and not T_FR_. While 3- to 4-fold less numerous than T_FH_, substantial numbers of follicular CD8 T-cells (fCD8 T-cells) infiltrated GC in acutely SIV-infected macaques, regardless BAFF blockade (Figure 7F). The extent of GC apoptosis was also comparable in both groups of macaques as shown by the quantification of active caspase-3 (aCasp-3) positive cells (Figure 7G). The number of apoptotic cells in GC correlated with that of PD1^+^ cells in IHC (Figure 7H) and a positive correlation was also found between the percentages of T_FH_ in mCD4 T-cells (in FCM) and the proportions of aCasp-3^+^ cells/mm^2^ of GC (Figure 7I). Thus, BAFF blockade does not impair the survival of T-cells or change the composition of the T-cell pool in GC.

### Functional Capacities of T_FH_ and Other mCD4 T-Cell Subsets in SIV-Infected Macaques

**Figure 8:**
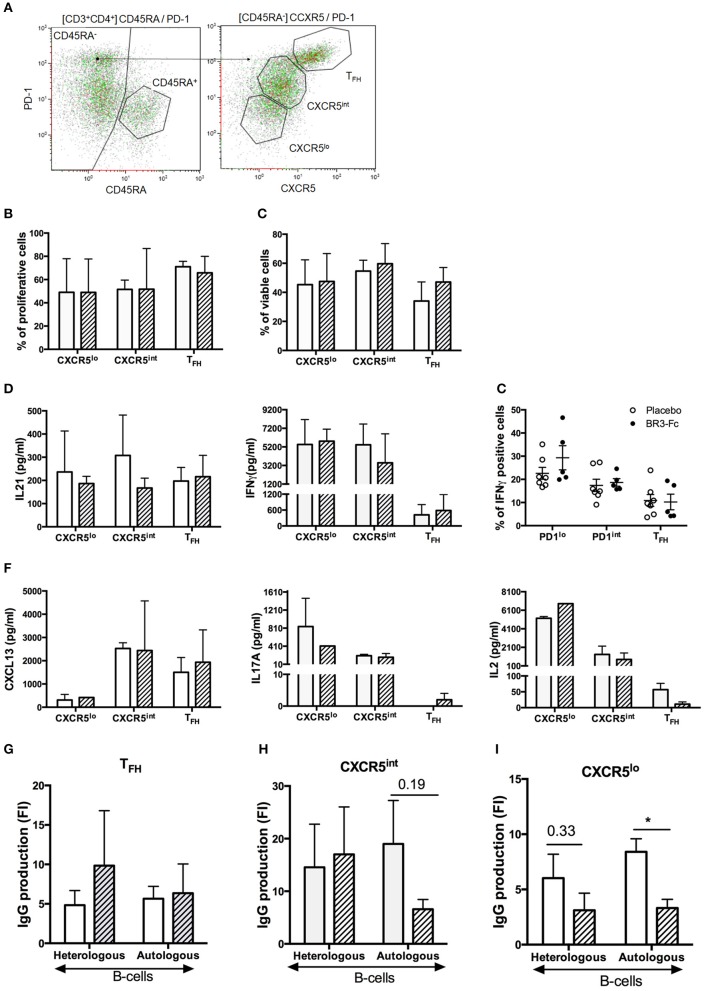
Sorting strategy and Functional responses of T_FH_ from Placebo and BR3-Fc treated macaques. **(A)** After gating on live CD3^+^CD4^+^CD45RA^−^ T-cells, mCD4 T-cells were sorted according to the intensity of PD1 and CXCR5 expression into three populations: T_FH_ (PD1^hi^CXCR5^hi^), CXCR5^int^ (PD1^int^CXCR5^int^), and CXCR5^lo^ (PD1^lo^CXCR5^lo^). Sorting was performed on spleen from 4 macaques of each group. Cells from each sorted subset were labeled with CSFE prior to culture with medium or CD3/2/28 stimulatory beads. On day 5, cell proliferation **(B)** and viability **(C)** were compared between groups. **(D)** Concentrations of IL21 and IFNγ in cell-free culture supernatants are shown. **(E)** Intracellular expression of IFNγ in spleen cells. Graph shows cumulative data obtained on 7 Placebo and 5 Treated animals. Each dot corresponds to one macaque of Placebo (*open circle*) and Treated (*black circle*) group. Bars represent mean ± SEM. **(F)** Concentrations of CXCL13, IL17A and IL2 in culture supernatants were quantified by Multiplex assay. **(G–I)** Sorted T-cells (10^4^/well) were cultured with heterologous or autologous B-cells (10^4^/well) in the presence of SEB for 7 days before quantifying IgG production in cell-free culture supernatant. Cultures of B-cells plus SEB were used as controls. Results are shown in fold increase (FI) calculated as the ratio between IgG value in co-culture with T-cells and SEB and IgG value in culture without T-cells (B-cells plus SEB). **(B–D,F–I)** Mean ± SEM are shown for Placebo (*open bar)* and Treated (*hatched bar)* group. For all panels, statistical comparison between groups was performed using the Mann Whitney non-parametric test. Significant statistical values are indicated *p < 0.05.

To assess the functional properties of T_FH_ and other mCD4 T-cell subsets in our 2 groups of SIV-infected macaques, we sorted mCD4 T-cells into three subsets according to the expression level of PD1 and CXCR5 [(Table S3, Panel 9) and Figure 8A]. As determined in preliminary experiments, similar distribution and proportions of T_FH_ and other mCD4 T-cell subsets were observed using Panels 8 and 9 (Figure S4). After sorting, functional features of mCD4 T-cell subsets were subsequently compared. Proliferation (Figure 8B) and survival (Figure 8C) in response to CD3/CD2/CD28 beads were similar for T_FH_ and the two other mCD4 T-cell subsets in both groups (Figures 8B,C). Addition of BAFF did not improve the survival of T_FH_ from Placebo or Treated macaques (*data not shown*).

We next measured IL21 and IFNγ concentration in supernatants and showed that T_FH_ from Placebo and Treated macaques released comparable amounts of IL21 (197 ± 29 vs. 216 ± 53 pg/ml, Figure 8D, left). Surprisingly, we also found consistent levels of IL21 produced by the other mCD4 T-cell subsets. In particular, IL21 concentration in the supernatants of CXCR5^int^ T-cells was 1.8-fold higher in Placebo than in Treated group (308 ± 100 vs. 167 ± 25 pg/ml). Consistent with type 1 helper T-cells being CXCR5 negative, IFNγ concentration was high in the supernatants of CXCR5^lo^ and CXCR5^int^ T-cell subsets. For this latter subset, IFNγ concentration tended to be higher in the supernatants from Placebo than from Treated macaques (5,424 ± 1,29 1pg/ml vs. 3,493 ± 1,796 pg/ml). IFNγ concentration was low in supernatants of T_FH_ with a slight increase in Treated macaques compared to Placebo (583 ± 349 vs. 420 ± 194 pg/ml). To further evaluate potential difference in IFNγ expression by T_FH_ between the two groups, we performed staining using ICOS and PD1 as surface markers to define the three subsets (Table S3, Panel 10). Compared to other fractions, the PD1^lo^ fraction contained elevated percentages of IFNγ^+^ cells (Figure 8E). Lower but comparable percentages of IFNγ^+^ cells were present in T_FH_ from both groups, with 11.9 ± 2.8 and 10.2 ± 3.3% positive cells in Placebo and Treated macaques, respectively (Figure 8E). Thus, BR3-Fc treatment did not alter IFNγ expression in T_FH_ of SIV-infected macaques.

By using Multiplex analysis, we also quantified CXCL13, BAFF, IL4, IL10, IL6, IL2, and IL17A in supernatants of various T-cell cultures (Figure 8F). In contrast to BAFF, IL4, IL6, and IL10 levels which remained lower than 20 pg/ml in all supernatants (*data not shown*), levels of CXCL13 were higher in supernatants of T_FH_ and CXCR5^int^ T-cells with no difference between the two groups of macaques. Concentrations of IL17A were below 10 pg/ml in supernatants of stimulated T_FH_ but expressed at higher but similar levels by CXCR5^int^ and CXCR5^lo^ supernatants from both groups. IL2 was highly produced by CXCR5^lo^ subsets and to a lesser extent by CXCR5^int^ subsets with similar levels in both groups. In T_FH_, average IL2 concentrations were lower than 30 pg/ml in both groups. Therefore, T_FH_ essentially produce CXCL13, IL21, and low levels of IFNγ at 28dpi but negligible levels of BAFF, IL17A, IL2, IL4, IL6, and IL10.

Finally, we compared the capacity of the various mCD4 T-cell subsets to sustain SEB-induced IgG production by autologous and/or heterologous B-cells purified from two healthy non-infected macaques. IgG production by heterologous and autologous B-cells was not significantly different in the presence of T_FH_ from both groups (Figure 8G). Surprisingly, IgG concentrations in cultures with CXCR5^int^ T-cells were higher or at least comparable (autologous B-cells, Treated group) to those observed in the presence of T_FH_ (Figure 8H) despite similar levels of IL21 and no IL6 and IL10 produced by those cells. Higher concentrations of IFNγ and IL2 might contribute to this difference. The reduced IgG production of autologous B-cells in the presence of CXCR5^int^ T-cells of Treated group compared to Placebo group might be a consequence of the combined reduction of IFNγ and IL21 concentrations as mentioned above. CXCR5^lo^ T-cells from Placebo group tended to be more efficient than those from Treated group at supporting IgG production by heterologous (2-fold, *p* = ns) and autologous (2.5-fold, *p* = 0.012) B-cells (Figure 8I).

## Discussion

During pathogenic HIV/SIV infection, GC concentrate infectious virus in T_FH_, T_FR_ and in long-lived immune complexes bound to FDC (67). To improve the early production of potent neutralizing Abs by reshaping the effector B-cell response, a better understanding of the GC reaction is required. Given that BAFF is a key cytokine for GC maintenance in mice (31, 32) but its excess favors autoimmunity (46, 47), we hypothesized that blockade of the BAFF excess reported during the acute phase of SIV infection (40) might “reset” the GC reaction and initiate a more effective virus-specific response. We therefore compared changes in GC and effector B-cells as well as in T_FH_ occurring in acutely SIV-infected macaques, treated or not by BR3-Fc, a BAFF antagonist. To the best of our knowledge, this is the first study to examine the role of BAFF in GC reaction and B-cell response in the context of viral infection.

Despite efficient BAFF blockade, counts (blood) and proportions (spleen and terminal ileum) of total CD4^+^ T-cells at 28 dpi as well as the dynamics of pVL over the 1st month of infection were comparable in BR3-Fc-treated and Placebo macaques. However, pro-viral load in PBMC was significantly higher at 10 dpi in BR3-Fc treated macaques than in Placebo but tended to be lower at 28 dpi in spleen and terminal ileum. The transient increase in pro-viral load in Treated macaques inversely correlated with levels of plasma IFNα and CXCL10. Regarding the potent antiviral effect of type I IFN, its transient decrease might facilitate SIV infection and/or replication at 10 dpi. No difference was otherwise observed in circulating CD4^+^ T-cell counts or activation at 10 dpi. Decrease in pDC counts (blood) and proportions (lymphoid organs) despite their sustained recruitment into lymphoid organs argues for BAFF blockade reinforcing SIV-induced pDC apoptosis in lymphoid organs during the acute phase of infection. It seems unlikely that BR3-Fc interferes with the binding of BAFF and the function of pDC since they do not express BAFF-R and TACI (68). Moreover, we found that CpG-A-induced IFNα production by isolated human pDC was similar regardless of the addition of BR3-Fc in culture (*data not shown*). Given that exaggerated monocyte activation occurs when TACI signaling is suppressed in murine (69) and human monocytes (70), BAFF blockade might potentiate the release of pro-apoptotic cytokines by SIV-activated myeloid cells in macaques, impairing pDC survival in tissues. This point deserves further investigation.

Consistent with BAFF being a key survival factor of B-cells (62, 71), BR3-Fc treatment aggravated the SIV-induced decrease of circulating naïve and MZ B-cells at 28 dpi compared to baseline values. Compared to Placebo, loss in spleen naïve B-cells was more pronounced in Treated macaques (59%, *p* = 0.007), possibly contributing to lower levels of plasma IgM from 14 dpi (Figure S5). In contrast to blood, proportions of spleen MZ B-cells were moderately affected by BAFF blockade, which is unexpected regarding their profound loss observed in mice deficient for BAFF or BAFF-R (72) and in healthy cynomolgus macaques treated with 20 mg/kg BR3-Fc for 18 weeks with an average of 37% loss of MZ B-cells (52). Considering that the proportions of spleen MZ B-cells are drastically reduced by SIV infection [(55) and this current work], we suggest that BAFF increase fails to counteract such loss during the acute phase of infection.

However, MZ B-cells are potentially responsive to BAFF since they expressed high levels of BAFF-R in Placebo and Treated macaques. In Placebo macaques, TACI concurrently expressed by MZ B-cells might mitigate ligand-induced BAFF-R signaling according to previous data in humans and mice (73, 74). As a consequence of BR3-Fc treatment, percentages of TACI^+^ cells were reduced in all B-cell subsets, and particularly in MZ B-cells and the three subsets of memory B-cells. In contrast, BAFF-R expression was preferentially increased on MZ and RM B-cells, suggesting that BAFF deprivation limits BAFF-R internalization or its TACI-dependent cleavage in these two subsets (75). Our data reveal that AM B-cells, and to a lesser extent TLM B-cells, expressed TACI in Placebo macaques while BAFF-R expression was lower in AM and TLM than in RM B-cells. This result agrees with previous data showing increased proportions of circulating CD21^lo^ mature B-cells in HIV viremic patients (76). Increased proportions of circulating B-cells being BAFF-R^lo^ and TACI^+^ are also present in atypical (CD21^lo^) memory B-cells of HIV-infected children compared to non-infected children (77). Whether TACI expression on AM and TLM B-cells is also observed in other infection or vaccine settings remains to be studied. Because TACI interacts with TLR9, its expression might potentiate the activation of atypical memory B cells (73).

Compared to Placebo, we did not observe major changes in splenic GC in BR3-Fc Treated macaques. Both the average size of GC and the proportions of GC B-cells among total B-cells were roughly comparable in both groups. However, a trend to lower proportions of proliferating cells was observed in spleen and terminal ileum of Treated macaques compared to Placebo, possibly indicating recycling defaults of GC B-cells between dark and light zones (78). BAFF neutralization failed to increase the recruitment of T_FR_ (FoxP3^+^ cells in GC), thought to ameliorate selection of virus-specific B-cells and affinity maturation (79) as well as to decrease fCD8 influx. However, these fCD8 cells endowed with regulatory functions during chronic infection (80) did not change IL21 release by T_FH_ during acute SIV infection. Similar proportions of T_FH_ among mCD4 T-cells and average numbers of spleen T_FH_ per GC in both groups of macaques argue for unchanged recruitment of T_FH_ despite BAFF deprivation. Of note, <2% of spleen T_FH_ (or either mCD4 T-cell subset) express BAFF-R or TACI in either group of SIV-infected macaques (Figure S6) and GC B-cells express BAFF-R at low level, with <5% GC B-cells being TACI^+^. Thus, it seems unlikely that BAFF directly affects spleen T_FH_ or GC B-cell expansion, at least in this setting. However, proportions of GC B-cells and T_FH_ concomitantly decreased by 4- and 2-fold in terminal ileum in the Treated group compared to Placebo, neither of these differences reaching statistical significance (Table S6). Thus, BAFF deprivation might have a more pronounced impact on mucosal than splenic GC reaction.

Contrasting with data of Goenka et al. in mice (36), sorted T_FH_ did not produce BAFF at 28 dpi, irrespective of BR3-Fc treatment. According to the kinetics of plasma BAFF in SIV-infected macaques, T_FH_ might only release BAFF transiently around 10 dpi (i.e., : at the peak of pVL). Release of IFNγ by sorted T_FH_ was 10-fold lower than that observed in other sorted mCD4^+^ T-cell subsets but remained comparable in both groups of SIV-infected macaques. This later result contrasts with those of Coquery et al. showing that BAFF enhances IFNγ production by human blood T_FH_ (35). In addition to IFNγ and IL21, we observed similar production of CXCL13 by sorted T_FH_ of both groups_._ However, PD1^int^ CXCR5^int^ CD4^+^ T-cells also released substantial levels of CXCL13 in both groups. IgG production by autologous and heterologous B-cells was similarly supported by T_FH_ of both groups but even more strongly by CXCR5^int^ T-cells. CXCL13 production and BCL6 expression by a fraction (21% in both groups) of CXCR5^int^ T-cells suggest that a fraction of them is T_FH_-related and possibly more efficient at supporting B-cell response than CXCR5^hi^ T_FH_.

Whereas CXCL13 was produced at similar levels by T_FH_ in both groups, SIV-induced CXCL13 plasma levels waned more rapidly in the BR3-Fc Treated group from 14 dpi (i.e., after the peak of pVL). This suggests that functions of FDC, key producers of CXCL13 and BAFF in GC (36, 81), are altered by BAFF blockade in SIV infection setting. Prior studies have consistently shown that maintenance of the GC reaction is reduced over time in BAFF deficient mice with a reduced network of mature FDC bearing less immune-complexes during response to TD antigen (31, 32). More recent data showing that human FDC lacks receptors for BAFF and that HIV does not directly bind to or signal in FDC (82) suggest an indirect impact of BAFF deprivation on FDC. One possibility is that BAFF deprivation in SIV-infected macaques reduces expression of membrane LTα1β2 on GC B-cells, limiting in turn FDC maturation. Alternatively, CXCL13 expression has been found in monocyte and immature DC of chronically HIV-infected patients (65) and can be induced in monocytes by pDC-released type I IFN upon HIV/SIV exposure (83). Regarding previous discussion on altered pDC renewal in BR3-Fc Treated macaques, decrease in circulating CXCL13 levels might be associated with this pDC loss in tissues.

Whereas, total circulating memory B-cells were 24% more numerous in Treated macaques than in Placebo at 28 dpi, the relative proportions of RM, AM, and TLM B-cell subsets remain unchanged (*data not shown*). In spleen and terminal ileum, increased proportions of total memory B-cells were observed in Treated macaques that preferentially benefit AM B-cells in spleen and RM B-cells in ileum. Consistent with more functional memory B-cells among spleen B-cells from Treated macaques, IgG production by spleen B-cells was significantly increased in culture with ligands of CD40 and TLR9 (CpG-B) and cytokines. These data clearly establish that BAFF blockade does not prevent the generation or survival of AM and TLM B-cells in GC during acute SIV infection. However, BAFF deprivation drastically reduces the surface expression of TACI on all memory B-cell subsets, possibly contributing to higher BAFF-R responsiveness in RM B-cells. Because a crosstalk between BAFF-R and BCR exists in mature B-cells (84), low BAFF-R expression in atypical memory B-cells might dampen their BCR signaling in SIV/HIV infection setting.

Despite similar frequencies of plasma blasts in GC, irrespective of treatment, plasma levels of SIV-specific Abs decreased in Treated macaques. Concurrently, plasma levels of anti-TT IgG also decreased while response against TT has been established prior to SIV infection. Collectively, these data suggest that BAFF deprivation reduces the survival of plasma cells. Given the reduced proportions of CD4^+^ T-cells during acute SIV infection, additional BAFF blockade might foster the destruction of plasma cells in niches according to Thai et al. (85). BAFF blockade might also reduce TACI expression on plasma blasts/cells, as it does on other B-cell subsets. Consistently, increased proportions of TACI^+^ BAFF-R^lo^ plasma blasts have been reported in blood of HIV-infected children compared to healthy children (77).

In conclusion, our work suggests that BAFF neutralization mainly affects B-cells and pDC during SIV infection. On one hand, BAFF neutralization might aggravate the SIV-induced death of pDC after their early recruitment into lymphoid organs. During the first 2 weeks of SIV infection where plasma viral load rapidly increases, reduced numbers of tissue pDC might participate in lower titers of type I IFN, contributing in turn to higher pro-viral load in PBMC and likely in tissues. On the other hand, BAFF neutralization impaired the survival of naïve and MZ B-cells to the benefit of memory B-cells without preventing the SIV-induced generation of atypical memory B-cells. Despite similar size of GC and frequency of GC B-cells, reduced frequency of proliferating GC B-cells was observed. This decrease could be due to either abnormal recycling of GC B-cells between the dark and light zones or to reduced number of naïve B-cells entering GC. This later hypothesis is consistent with reduced CXCL13 levels produced by FDC following cognate interactions with B-cells. Moreover, BAFF blockade impaired the survival of plasma cells as shown by decreased titers of both SIV- and TT-specific antibodies. Lastly, this work described the expression of BAFF receptors in spleen B-cells and T_FH_ during SIV infection.

## Data Availability Statement

All datasets generated for this study are included in the article/Supplementary Material.

## Ethics Statement

The animal study was reviewed and approved by the Ministère de l'Education Nationale, de l'Enseignement Supérieur et de la Recherche (France) and the Comité d'Ethique en Expérimentation Animale n°44 under the reference 2015121509045664 (APAFIS#3178).

## Author Contributions

GB, MT, HI, and SI performed experiments, acquired data, and contributed to data analysis. NB and RL supervised animal study management and viral load quantification. GB, MT, HI, SI, and YR discussed results and contributed to the writing of the manuscript. YR designed the study, obtained funding, provided study oversight, analyzed data, and drafted this manuscript. All authors contributed to manuscript revision and approved it.

### Conflict of Interest

The authors declare that the research was conducted in the absence of any commercial or financial relationships that could be construed as a potential conflict of interest.
