# Supplementary material for: Impact of BAFF Blockade on Inflammation, Germinal Center Reaction and Effector B-Cells During Acute SIV Infection
Source: Front Immunol. 2020 Feb 28;11:252. doi: 10.3389/fimmu.2020.00252 (PMC7061218; doi:10.3389/fimmu.2020.00252)
Supplement: Supplementary file 1 [file Table_1.DOCX]

|  |
| --- |

**Table S1. Genotypes of SIV-infected macaques**

| Placebo | BR3-Fc |
| --- | --- |
| H2/H1 | H2/H1 |
| H2/RecH1H4 | H2/RecH1H5 |
| H2/H4 | RecH2H1/H5 |
| RecH3H2/RecH2H1 | H3/RecH2H1 |
| H3/RecH1H5 | H3/RecH1H5 |
| H3/H5 | H3/H5 |
| H3/H3 |  |
| H3/H1 |  |
| H3/H2 |  |
| H3/H1 |  |

|  |
| --- |
