# Supplementary material for: Impact of BAFF Blockade on Inflammation, Germinal Center Reaction and Effector B-Cells During Acute SIV Infection
Source: Front Immunol. 2020 Feb 28;11:252. doi: 10.3389/fimmu.2020.00252 (PMC7061218; doi:10.3389/fimmu.2020.00252)
Supplement: Supplementary file 2 [file Table_2.DOCX]

**Table S2. Antibody panels for multiparameter flow cytometry**

| **Panel 1** | | | | **Panel 2** | | | | **Panel 3** | | | | |  |  |
| --- | --- | --- | --- | --- | --- | --- | --- | --- | --- | --- | --- | --- | --- | --- |
| **Leukocyte subpopulations** | | | | **Monocyte & DC subtypes** | | | | **Plasmacytoid DC recruitment** | | | | |  |  |
| *Fluorescent dye* | *Antigen* | *clone* | *Manufact^a^* | *Fluorescent dye* | *Antigen* | *clone* | *Manufact* | *Fluorescent dye* | *Antigen* | | *clone* | *Manufact* |  |  |
| Blue stain (UV) | **Live-Dead** | / | Invitrogen | Blue stain (UV) | **Live-Dead** | / | Invitrogen | Blue stain (UV) | **Live-Dead** | | / | Invitrogen |  |  |
| PerCP | **CD45** | D058-1283 | BD | PerCP | **CD45** | D058-1283 | BD | PerCP | **CD45** | | D058-1283 | BD |  |  |
| V500 | **CD3** | SP34.2 | BD | V500 | **CD3** | SP34.2 | BD | V500 | **CD3** | | SP34.2 | BD |  |  |
| AlexaFluor700 | **CD20** | 2H7 | BLE | AlexaFluor700 | **CD20** | 2H7 | BLE | AlexaFluor700 | **CD20** | | 2H7 | BLE |  |  |
| Pe-Cy7 | **CD4** | L200 | BD | Pe-Cy7 | **CD14** | M5E2 | BD | Pe-Cy7 | **CD14** | | M5E2 | BD |  |  |
| Vioblue | **CD8** | BW135/80 | MACS | APC-H7 | **CD16** | 3G8 | BD | Vioblue | **CD8** | | BW135/80 | MACS |  |  |
| APC | **CD14** | M5E2 | BD | ECD | **HLA-DR** | Immu357 | Coulter | PE | **CD123** | | 7G3 | BD |  |  |
|  |  |  |  | FITC | **BDCA-2** | AC144 | MACS | APC-H7 | **HLA-DR** | | L243 | BD |  |  |
|  |  |  |  | PE | **CD123** | 7G3 | BD | AlexaFluor488 | **Ki67** | | B56 | BD |  |  |
|  |  |  |  | APC | **CD1c** | AD5-8E7 | MACS | AlexaFluor488 | **mIgG1** | | MOPC21 | BD |  |  |
|  |  |  |  | APC | **mIgG2a** | S43.10 | MACS |  |  | |  |  |  |  |
| **Panel 4** | | | | | **Panel 5** | | | |  | | | | | |
| **B-cell subsets in blood** | | | | **B-cell subsets in lymphoid organs** | | | |  | | | | |  |  |
| *Fluorescent dye* | *Antigen* | *clone* | *Manufact* | *Fluorescent dye* | *Antigen* | *clone* | *Manufact* |  |  |  | |  |  |  |
| Blue stain (UV) | **Live-Dead** | / | Invitrogen | Blue stain (UV) | **Live-Dead** | / | Invitrogen |  |  |  | |  |  |  |
| PerCP | **CD45** | D058-1283 | BD | PerCP | **CD45** | D058-1283 | BD |  |  |  | |  |  |  |
| V500 | **CD3** | SP34.2 | BD | V500 | **CD3** | SP34.2 | BD |  |  |  | |  |  |  |
| AlexaFluor700 | **CD20** | 2H7 | BLE | AlexaFluor700 | **CD20** | 2H7 | BLE |  |  |  | |  |  |  |
| Pe-Cy7 | **CD19** | J3.119 | Coulter | Pe-Cy7 | **CD19** | J3.119 | Coulter |  |  |  | |  |  |  |
| FITC | **IgD** | Rabbit IgG | AbD | FITC | **IgD** | Rabbit IgG | AbD |  |  |  | |  |  |  |
| BV711 | **CD21** | B-ly4 | BD | BV711 | **CD21** | B-ly4 | BD |  |  |  | |  |  |  |
| PE | **CD27** | M-T271 | BD | PE | **CD27** | M-T271 | BD |  |  |  | |  |  |  |
|  |  |  |  | AlexaFluor647 | **Bcl-6** | K112-91 | BD |  |  |  | |  |  |  |
|  |  |  |  | AlexaFluor647 | **mIgG1** | MOPC21 | BD |  |  |  | |  |  |  |
|  |  |  |  | BV421 | **Ki67** | B56 | BD |  |  |  | |  |  |  |
|  |  |  |  | BV421 | **mIgG1** | X40 | BD |  |  |  | |  |  |  |

(a) Manufacturer/distributor - **AbD**: AbD Serotec - Bio-Rad, Colmar, France; **BD**: BD Biosciences, Rungis, France; **BLE**: Biolegend, Ozyme, St Quentin-en-Yvelines, France**; Coulter**: Beckman Coulter, Villepinte, France; **Invitrogen**: Life Technologies SAS, Saint Aubin, France; **MACS**: Miltenyi Biotec –Paris, France.
