# Supplementary material for: Impact of BAFF Blockade on Inflammation, Germinal Center Reaction and Effector B-Cells During Acute SIV Infection
Source: Front Immunol. 2020 Feb 28;11:252. doi: 10.3389/fimmu.2020.00252 (PMC7061218; doi:10.3389/fimmu.2020.00252)
Supplement: Supplementary file 3 [file Table_3.DOCX]

**Table S3. Antibody panels for the detection of BAFF receptors and T_FH_ by flow cytometry**

| **Panel 6** | | | | | **Panel 7** | | | | **Panel 8** | | | | |
| --- | --- | --- | --- | --- | --- | --- | --- | --- | --- | --- | --- | --- | --- |
| **BAFF-R expression on B-cells** | | | | | **TACI expression on B-cells** | | | | **Follicular Helper T-cells** | | | | |
| *Fluorescent dye* | | *Antigen* | *clone* | *Manufact^a^* | *Fluorescent dye* | *Antigen* | *clone* | *Manufact* | *Fluorescent dye* | *Antigen* | | *clone* | *Manufact^a^.* |
| Blue stain (UV) | | **Live-Dead** | / |  | Blue stain (UV) | **Live-Dead** | / | Invitrogen | Blue stain (UV) | **Live-Dead** | | / | Invitrogen |
| PerCP | | **CD45** | D058-1283 | BD | PerCP | **CD45** | D058-1283 | BD | PerCP | **CD45** | | D058-1283 | BD |
| V500 | | **CD3** | SP34.2 | BD | V500 | **CD3** | SP34.2 | BD | V500 | **CD3** | | SP34.2 | BD |
| Pe-Cy7 | | **CD19** | J3.119 | Coulter | Pe-Cy7 | **CD19** | J3.119 | Coulter | Pe-Cy7 | **CD4** | | L200 | BD |
| AlexaFluor700 | | **CD20** | 2H7 | BLE | AlexaFluor700 | **CD20** | 2H7 | BLE | APC-H7 | **CD45RA** | | 5H9 | BD |
| FITC | | **IgD** | Rabbit IgG | AbD | FITC | **IgD** | Rabbit IgG | AbD | PE | **ICOS** | | C398.4A | BLE |
| BV711 | | **CD21** | B-ly4 | BD | BV711 | **CD21** | B-ly4 | BD | BV711 | **PD-1** | | EH12.2H7 | BLE |
| BV421 | | **CD27** | M-T271 | BD | BV421 | **CD27** | M-T271 | BD | AlexaFluor647 | **Bcl-6** | | K112-91 | BD |
| PE | | **BAFF-R** | 11C1 | BLE | PE | **TACI** | 1A1 | BLE | AlexaFluor647 | **mIgG1** | | MOPC21 | BD |
| PE | | **mIgG1** | MOPC21 | BLE | PE | **Rat IgG2a** | RTK2758 | BLE | BV421 | **Ki67** | | B56 | BD |
| AlexaFluor647 | | **Bcl-6** | K112-91 | BD | AlexaFluor647 | **Bcl-6** | K112-91 | BD | BV421 | **mIgG1** | | X40 | BD |
| AlexaFluor647 | | **mIgG1** | MOPC21 | BD | AlexaFluor647 | **mIgG1** | MOPC21 | BD |  |  | |  |  |
|  | | | |  | | | |  | | | | | |
| **Panel 9** | | | | **Panel 10** | | | |  | | | | | |
| **Follicular helper T-cells (Sorting)** | | | | | **IFNγ-producing T cells** | | | |  | | | | |
| *Fluorescent dye* | | *Antigen* | *clone* | *Manufact* | *Fluorescent dye* | *Antigen* | *clone* | *Manufact* |  |  |  | |  |
| Yellow stain (UV) | | **Live-Dead** | / | Invitrogen | Blue stain (UV) | **Live-Dead** | / | Invitrogen |  |  |  | |  |
| AlexaFluor488 | | **CD3** | SP34.2 | BD | PerCP | **CD45** | D058-1283 | BD |  |  |  | |  |
| Pe-Cy7 | | **CD4** | L200 | BD | V500 | **CD3** | SP34.2 | BD |  |  |  | |  |
| APC-H7 | | **CD45RA** | 5H9 | BD | Pe-Cy7 | **CD4** | L200 | BD |  |  |  | |  |
| PE | | **ICOS** | C398.4A (REA) | MACS | Vioblue | **CD8** | BW135/80 | MACS |  |  |  | |  |
| BV711 | | **PD-1** | EH12.2H7 | BLE | APC-H7 | **CD45RA** | 5H9 | BD |  |  |  | |  |
| APC | | **CXCR5** | MU5UBEE | BLE | AlexaFluor700 | **IFNγ** | 4SB3 | BLE |  |  |  | |  |
|  | |  |  |  | AlexaFluor700 | **mIgG1** | MOPC21 | BLE |  |  |  | |  |

(a) Manufacturer/distributor - **AbD**: AbD Serotec - Bio-Rad, Colmar, France; **BD**: BD Biosciences, Rungis, France; **BLE**: Biolegend, Ozyme, St Quentin-en-Yvelines, France**; Coulter**: Beckman Coulter, Villepinte, France; **Invitrogen**: Life Technologies SAS, Saint Aubin, France; **MACS**: Miltenyi Biotec –Paris, France.
