# Supplementary material for: Impact of BAFF Blockade on Inflammation, Germinal Center Reaction and Effector B-Cells During Acute SIV Infection
Source: Front Immunol. 2020 Feb 28;11:252. doi: 10.3389/fimmu.2020.00252 (PMC7061218; doi:10.3389/fimmu.2020.00252)
Supplement: Supplementary file 4 [file Table_4.DOCX]

**Table S4. Antibodies for Immunohistochemistry**

| Antibodies | Clone | Species (Isotype) | Manufacturer^a^ |
| --- | --- | --- | --- |
| CD20 | L26 | Mouse (IgG2a) | DAKO |
| CD3 |  | Rabbit (IgG) | DAKO |
| CD4 | 1F6 | Mouse (IgG1) | Novocastra |
| CD8 |  | Rabbit (IgG) | Abcam |
| FoxP3 | 259D | Mouse (IgG1) | BLE |
| Active Caspase-3 |  | Rabbit (IgG) | CST |
| Granzyme B | GrB7 | Mouse (IgG2a) | DAKO |
| Ki67 | B56 | Mouse (IgG1) | BD |
| IRF4 | MUM1 | Mouse (IgG1) | DAKO |
| IgM |  | Rabbit (F(ab)’_2_ IgG) | DAKO |
| IgG |  | Rabbit (F(ab)’_2_ IgG) | DAKO |
| PD1 |  | Goat (IgG) | Bio-techne |

**(a) Abcam**: Paris, France; **BLE**: Biolegend, Ozyme, St Quentin-en-Yvelines, France; **BD**: BD Biosciences, Rungis, France ; **Bio-techne**, Lille, France ; **CST**: Cell Signaling Technology, Ozyme, St Quentin-en-Yvelines, France; **DAKO**: Les Ulis, France; **Novocastra**: Leica Biosystems, Nanterre, France.
