# Supplementary material for: Impact of BAFF Blockade on Inflammation, Germinal Center Reaction and Effector B-Cells During Acute SIV Infection
Source: Front Immunol. 2020 Feb 28;11:252. doi: 10.3389/fimmu.2020.00252 (PMC7061218; doi:10.3389/fimmu.2020.00252)
Supplement: Supplementary file 5 [file Table_5.DOCX]

|  |
| --- |

**Table S5. B-cells in spleen of uninfected macaques**

Proportions of the various B-cell subsets were determined by FCM in spleens of six uninfected macaques.

| B-cell subset | n | MEAN | SEM |  |
| --- | --- | --- | --- | --- |
| Total | 6 | 44.5 | 3.5 | *% of CD45^+^ cells* |
| MZ | 6 | 25.7 | 5.2 | *% of total B-cells* |
| Naive | 6 | 40.5 | 5.2 | *% of total B-cells* |
| GC | 6 | 7 | 1.1 | *% of total B-cells* |
| Total Memory | 6 | 15.7 | 3.3 | *% of total B-cells* |
| RM | 6 | 7.1 | 1.5 | *% of total B-cells* |
| AM | 6 | 5.9 | 1.2 | *% of total B-cells* |
| TLM | 6 | 5.1 | 1 | *% of total B-cell* |
