# Supplementary material for: Impact of BAFF Blockade on Inflammation, Germinal Center Reaction and Effector B-Cells During Acute SIV Infection
Source: Front Immunol. 2020 Feb 28;11:252. doi: 10.3389/fimmu.2020.00252 (PMC7061218; doi:10.3389/fimmu.2020.00252)
Supplement: Supplementary file 6 [file Table_6.DOCX]

| **TABLE S6. Distribution of various subsets in terminal ileum of Treated and Placebo macaques** | | | | | | | | | | | | | | | |
| --- | --- | --- | --- | --- | --- | --- | --- | --- | --- | --- | --- | --- | --- | --- | --- |
|  |  |  |  | **Uninfected**  **(SIV^-^)^a^** | |  | **Placebo**  **(Plac.)** | | ***P***  ***values*** |  | **BR3-Fc** | | ***P values*** | ***P values*** |  |
|  |  |  |  | Mean | SEM |  | Mean | SEM | vs.SIV^-^ |  | Mean | SEM | vs. SIV^-^ | vs. Plac |  |
| **Proviral Load** | | |  | NA^b^ |  |  | 2440 | 690 | NA |  | 1804 | 296 | NA | ns^c^ |  |
|  |  |  |  |  |  |  |  |  |  |  |  |  |  |  |  |
| **pDC** | |  |  | 2.9 | 0.1 |  | 3.8 | 1.8 | ns^d^ |  | 3 | 0.7 | ns | ns | *% of Lin^-^DR+* |
|  |  | **Ki67^+^** |  | 2.8 | 0.6 |  | 17.7 | 2 | *** |  | 15.7 | 4.2 | ns | ns | *% of pDC* |
|  |  |  |  |  |  |  |  |  |  |  |  |  |  |  |  |
| **CD3^+^** |  |  |  | 41.1 | 12.3 |  | 43.5 | 4.9 | ns |  | 55.3 | 2.2 | ns | ns | *% of CD45^+^ cells* |
|  | **CD4^+^** |  |  | 64.1 | 1.9 |  | 10.4 | 1.9 | *** |  | 9.8 | 2.2 | *** | ns | *% of T-cells* |
|  |  | **Ki67^+^** |  | 7.4 | 0.7 |  | 25.1 | 5.9 | **^e^ |  | 16.8 | 1.7 | ns | ns | *% of CD4^+^ T-cells* |
|  |  | **T_FH_** |  | 8.1 | 1.9 |  | 13.7 | 3 | ns |  | 7.3 | 1.9 | ns | ns | *% of CD4+ T-cells* |
|  |  | **mCD4** |  | 89.5 | 0.8 |  | 67.1 | 3.9 | ***** |  | 69.7 | 5.1 | **** | ns | *% of CD4^+^ T-cells* |
|  |  |  | **T_FH_** | 9.1 | 2.2 |  | 20.6 | 4.9 | ns |  | 10.8 | 3.1 | ns | ns | *% of mCD4 T-cells* |
|  |  |  |  |  |  |  |  |  |  |  |  |  |  |  |  |
| **CD19^+^** |  |  |  | 55.4 | 12.5 |  | 51.4 | 3.4 | ns |  | 39.4 | 2,4 | ns | ns | *% of CD45^+^ cells* |
|  | **CD20^-^** |  |  | 5.5 | 2.2 |  | 4.3 | 0.9 | ns |  | 6.6 | 1.1 | ns | ns | *% of CD19^+^* |
|  | **CD20^+^** |  |  | 94.5 | 2.2 |  | 95.2 | 1 | ns |  | 93.4 | 1.1 | ns | ns | *% of CD19^+^* |
|  |  | **MZ** |  | 4.3 | 2.4 |  | 3.5 | 1.7 | ns |  | 2.4 | 0.7 | ns | ns | *% of CD20^+^* |
|  |  | **Naive** |  | 7.4 | 0.4 |  | 17.1 | 4.2 | ns |  | 7.3 | 2.8 | ns | ns | *% of CD20^+^* |
|  |  | **Tot Mem.** |  | 75.4 | 3.7 |  | 66.1 | 7.4 | ns |  | 81.4 | 4.2 | ns | ** | *% of CD20^+^* |
|  |  | **RM** |  | 69.8 | 3.8 |  | 61.3 | 7.9 | ns |  | 76.5 | 5.2 | ns | ** | *% of CD20^+^* |
|  |  | **AM** |  | 3.2 | 1.6 |  | 2.5 | 1 | ns |  | 2.3 | 0.5 | ns | ns | *% of CD20^+^* |
|  |  | **TLM** |  | 2.4 | 0.9 |  | 2.2 | 0.7 | ns |  | 2.6 | 0.9 | ns | ns | *% of CD20^+^* |
|  |  | **GC** |  | 6.3 | 2.1 |  | 9.2 | 4 | ns |  | 2.05 | 0.5 | ns | ns | *% of CD20^+^* |
|  |  |  | **Ki67^+^** | 72.5 | 4.1 |  | 78.2 | 8.3 | ns |  | 54.7 | 6.8 | ns | ns | *% of GC* |
|  |  |  |  |  |  |  |  |  |  |  |  |  |  |  |  |
| \| 1. Panels were tested on samples from 3 uninfected, 4 to 6 Placebo and 5 to 6 BR3-Fc treated macaques depending on available cell numbers. 2. NA: not applicable 3. Proviral loads were compared using a Mann Whitney test. *p* value is indicated in red. ns: not significant 4. Values of each group were compared using a (non parametric) Kruskal-Wallis test with Dunns’ s multiple comparisons test. *p* values are indicated in black. ns: not significant, *p<0.05 5. Proportions of various subsets in CD4^+^ T-cells and in CD20^+^ B-cells were compared using a 2-way ANOVA with Tukey’s multiple comparisons test. *p* values are indicated in blue. ns: not significant, ** p<0.01, ***p<0.001 \| \| --- \| | | | | | | | | | | | | | | | |
