# Supplementary material for: Impact of BAFF Blockade on Inflammation, Germinal Center Reaction and Effector B-Cells During Acute SIV Infection
Source: Front Immunol. 2020 Feb 28;11:252. doi: 10.3389/fimmu.2020.00252 (PMC7061218; doi:10.3389/fimmu.2020.00252)
Supplement: Supplementary file 7 [file Presentation_1.pptx]

## Slide 1
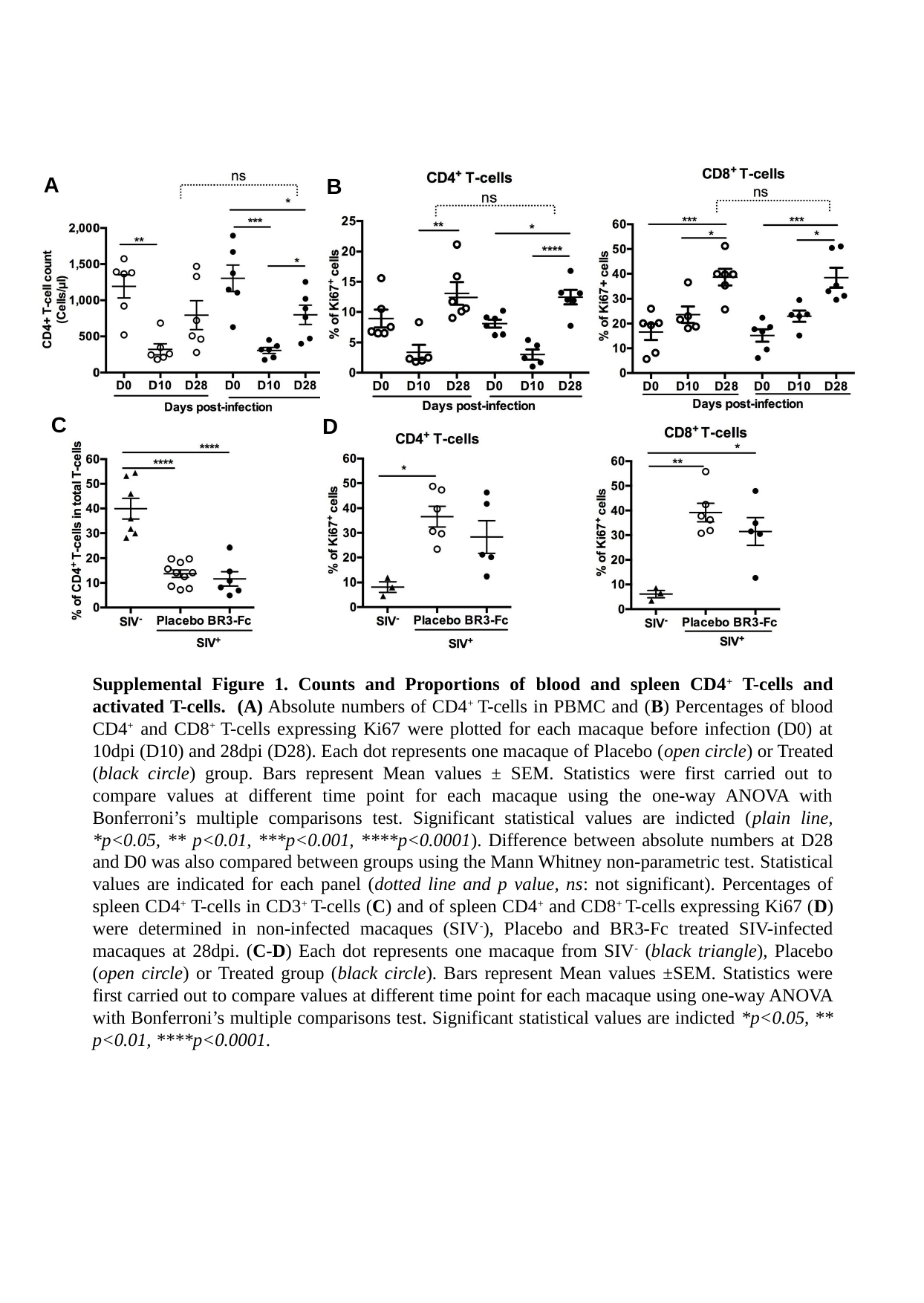

A
B
C
D
Supplemental Figure 1. Counts and Proportions of blood and spleen CD4+ T-cells and activated T-cells. (A) Absolute numbers of CD4+ T-cells in PBMC and (B) Percentages of blood CD4+ and CD8+ T-cells expressing Ki67 were plotted for each macaque before infection (D0) at 10dpi (D10) and 28dpi (D28). Each dot represents one macaque of Placebo (open circle) or Treated (black circle) group. Bars represent Mean values ± SEM. Statistics were first carried out to compare values at different time point for each macaque using the one-way ANOVA with Bonferroni’s multiple comparisons test. Significant statistical values are indicted (plain line, *p<0.05, ** p<0.01, ***p<0.001, ****p<0.0001). Difference between absolute numbers at D28 and D0 was also compared between groups using the Mann Whitney non-parametric test. Statistical values are indicated for each panel (dotted line and p value, ns: not significant). Percentages of spleen CD4+ T-cells in CD3+ T-cells (C) and of spleen CD4+ and CD8+ T-cells expressing Ki67 (D) were determined in non-infected macaques (SIV-), Placebo and BR3-Fc treated SIV-infected macaques at 28dpi. (C-D) Each dot represents one macaque from SIV- (black triangle), Placebo (open circle) or Treated group (black circle). Bars represent Mean values ±SEM. Statistics were first carried out to compare values at different time point for each macaque using one-way ANOVA with Bonferroni’s multiple comparisons test. Significant statistical values are indicted *p<0.05, ** p<0.01, ****p<0.0001.
