# Supplementary material for: Impact of BAFF Blockade on Inflammation, Germinal Center Reaction and Effector B-Cells During Acute SIV Infection
Source: Front Immunol. 2020 Feb 28;11:252. doi: 10.3389/fimmu.2020.00252 (PMC7061218; doi:10.3389/fimmu.2020.00252)
Supplement: Supplementary file 8 [file Presentation_2.pptx]

## Slide 1
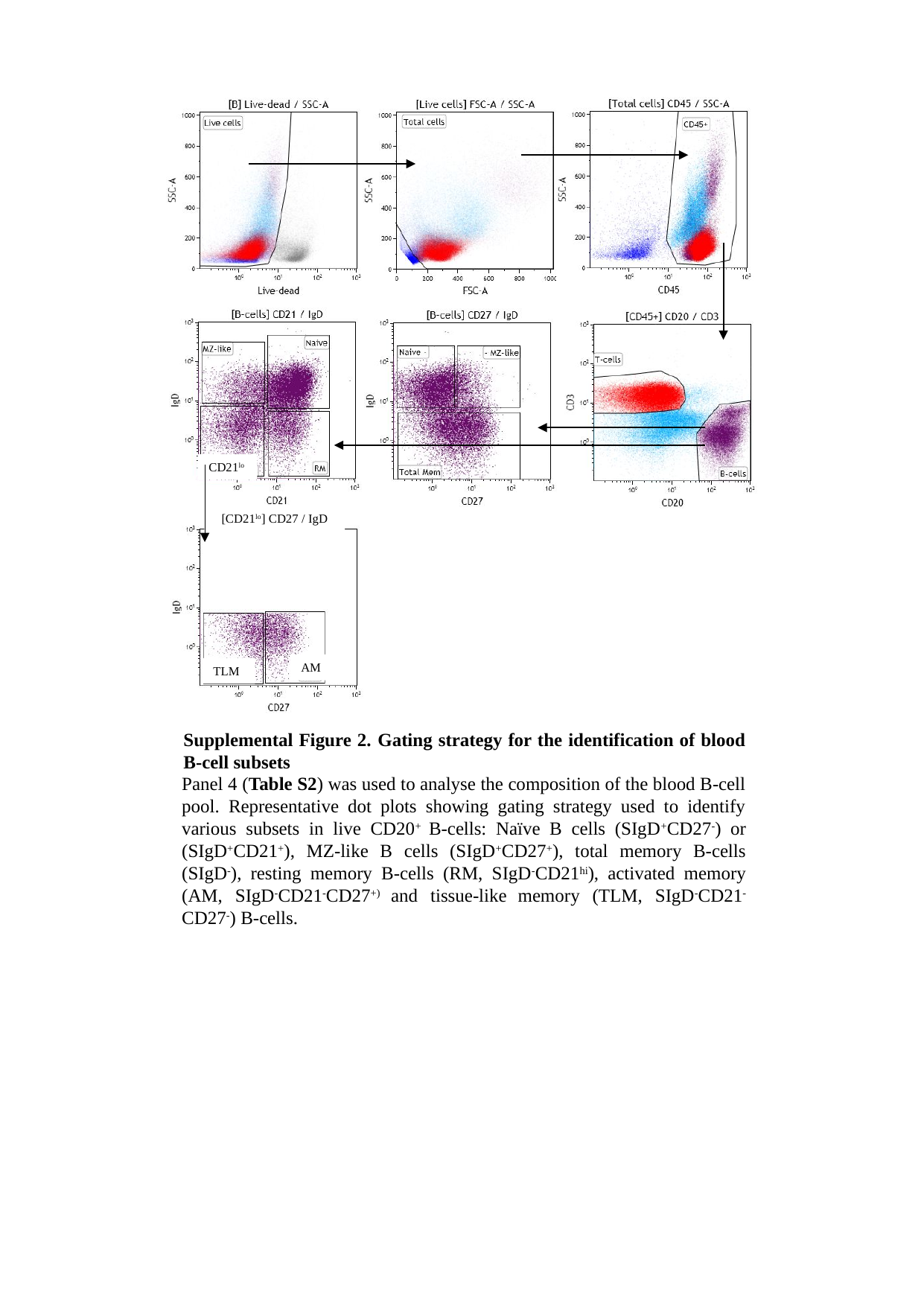

CD21lo
[CD21lo] CD27 / IgD
AM
TLM
Supplemental Figure 2. Gating strategy for the identification of blood B-cell subsets
Panel 4 (Table S2) was used to analyse the composition of the blood B-cell pool. Representative dot plots showing gating strategy used to identify various subsets in live CD20+ B-cells: Naïve B cells (SIgD+CD27-) or (SIgD+CD21+), MZ-like B cells (SIgD+CD27+), total memory B-cells (SIgD-), resting memory B-cells (RM, SIgD-CD21hi), activated memory (AM, SIgD-CD21-CD27+) and tissue-like memory (TLM, SIgD-CD21-CD27-) B-cells.
