# Supplementary material for: Impact of BAFF Blockade on Inflammation, Germinal Center Reaction and Effector B-Cells During Acute SIV Infection
Source: Front Immunol. 2020 Feb 28;11:252. doi: 10.3389/fimmu.2020.00252 (PMC7061218; doi:10.3389/fimmu.2020.00252)
Supplement: Supplementary file 9 [file Presentation_3.pptx]

## Slide 1
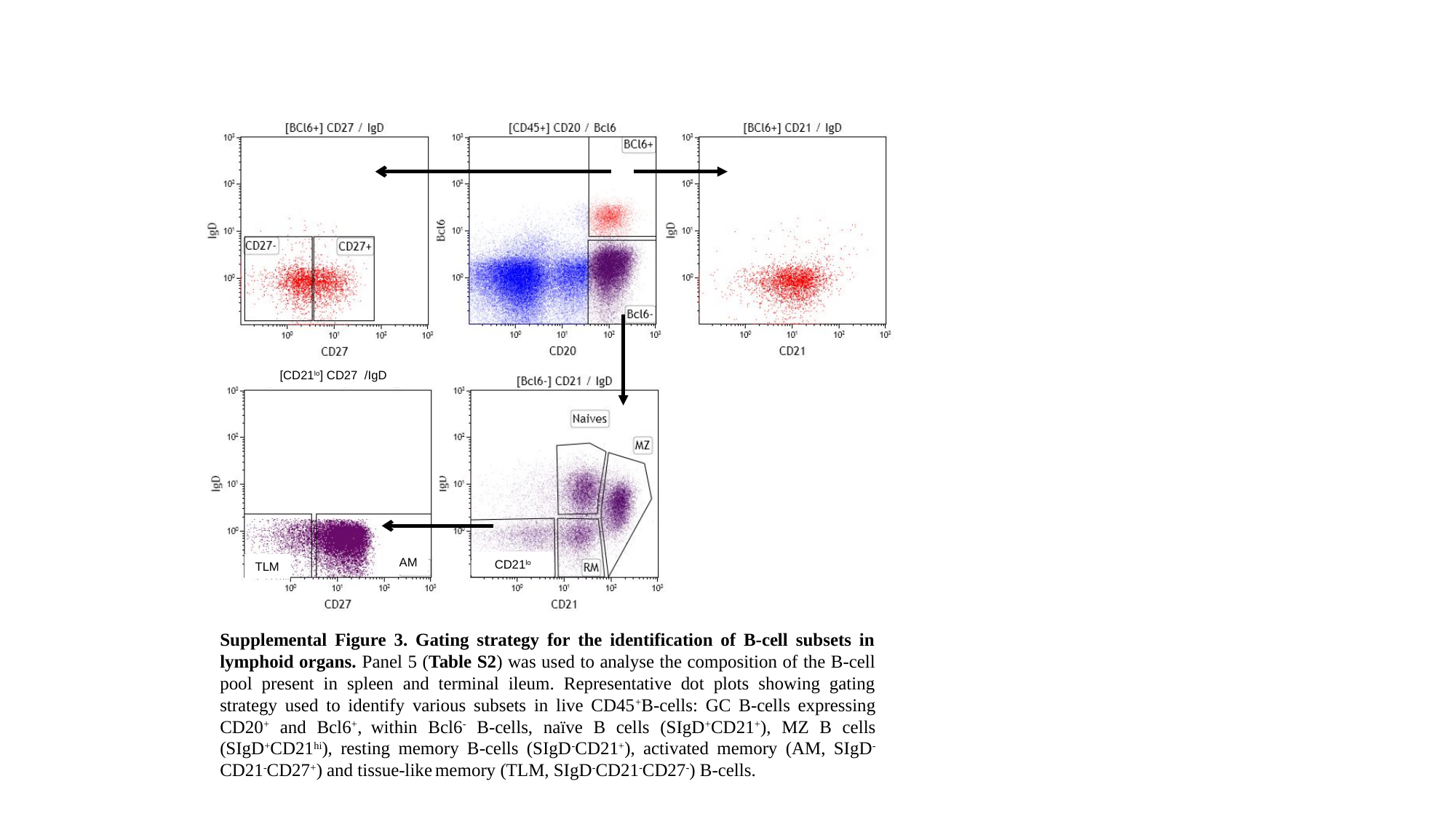

[CD21lo] CD27 /IgD
AM
TLM
CD21lo
Supplemental Figure 3. Gating strategy for the identification of B-cell subsets in lymphoid organs. Panel 5 (Table S2) was used to analyse the composition of the B-cell pool present in spleen and terminal ileum. Representative dot plots showing gating strategy used to identify various subsets in live CD45+B-cells: GC B-cells expressing CD20+ and Bcl6+, within Bcl6- B-cells, naïve B cells (SIgD+CD21+), MZ B cells (SIgD+CD21hi), resting memory B-cells (SIgD-CD21+), activated memory (AM, SIgD-CD21-CD27+) and tissue-like memory (TLM, SIgD-CD21-CD27-) B-cells.
