# Supplementary material for: Impact of BAFF Blockade on Inflammation, Germinal Center Reaction and Effector B-Cells During Acute SIV Infection
Source: Front Immunol. 2020 Feb 28;11:252. doi: 10.3389/fimmu.2020.00252 (PMC7061218; doi:10.3389/fimmu.2020.00252)
Supplement: Supplementary file 10 [file Presentation_4.pptx]

## Slide 1
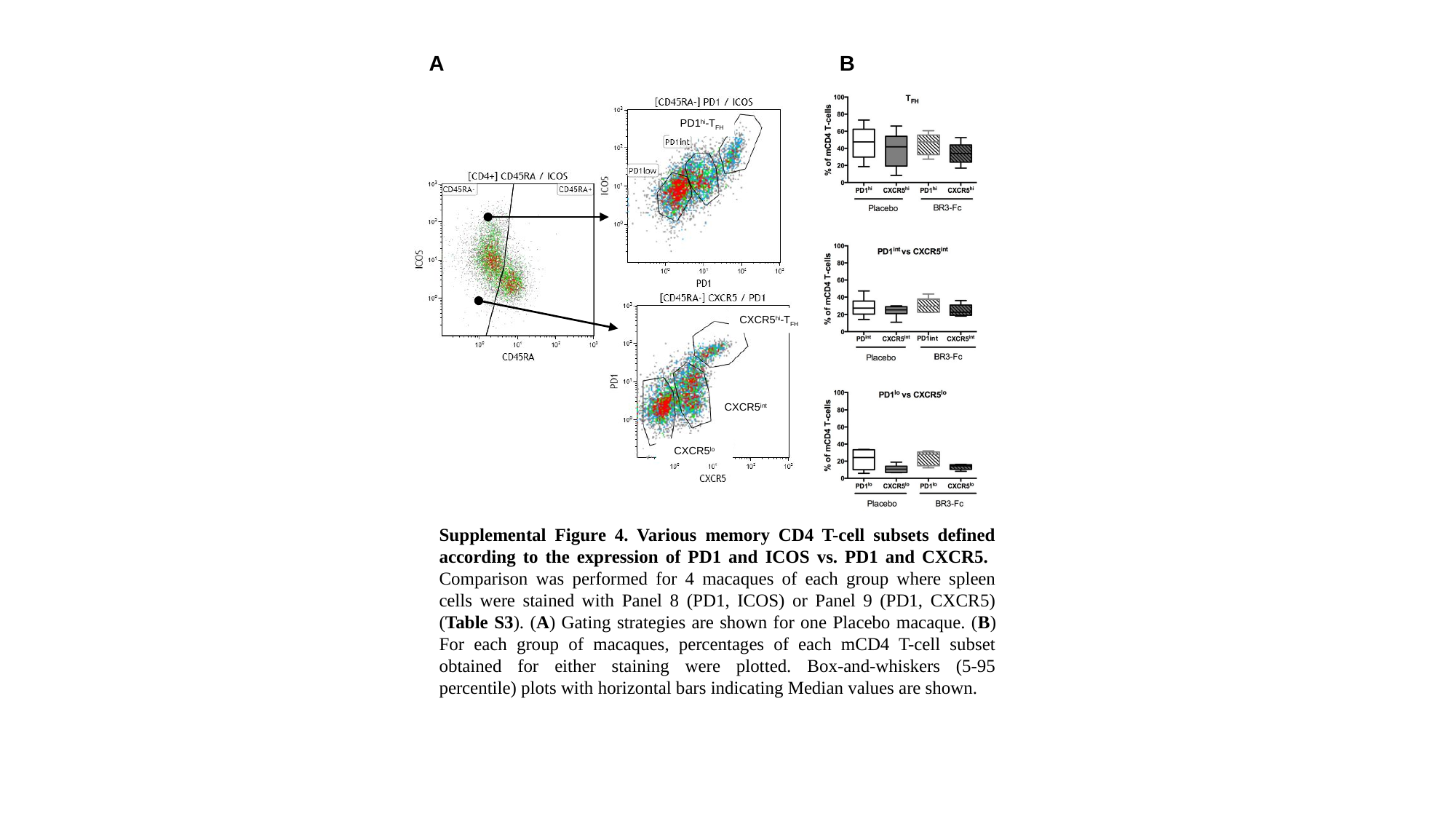

B
A
PD1hi-TFH
CXCR5hi-TFH
CXCR5int
CXCR5lo
Supplemental Figure 4. Various memory CD4 T-cell subsets defined according to the expression of PD1 and ICOS vs. PD1 and CXCR5. Comparison was performed for 4 macaques of each group where spleen cells were stained with Panel 8 (PD1, ICOS) or Panel 9 (PD1, CXCR5) (Table S3). (A) Gating strategies are shown for one Placebo macaque. (B) For each group of macaques, percentages of each mCD4 T-cell subset obtained for either staining were plotted. Box-and-whiskers (5-95 percentile) plots with horizontal bars indicating Median values are shown.
