# Supplementary material for: Impact of BAFF Blockade on Inflammation, Germinal Center Reaction and Effector B-Cells During Acute SIV Infection
Source: Front Immunol. 2020 Feb 28;11:252. doi: 10.3389/fimmu.2020.00252 (PMC7061218; doi:10.3389/fimmu.2020.00252)
Supplement: Supplementary file 11 [file Presentation_5.pptx]

## Slide 1
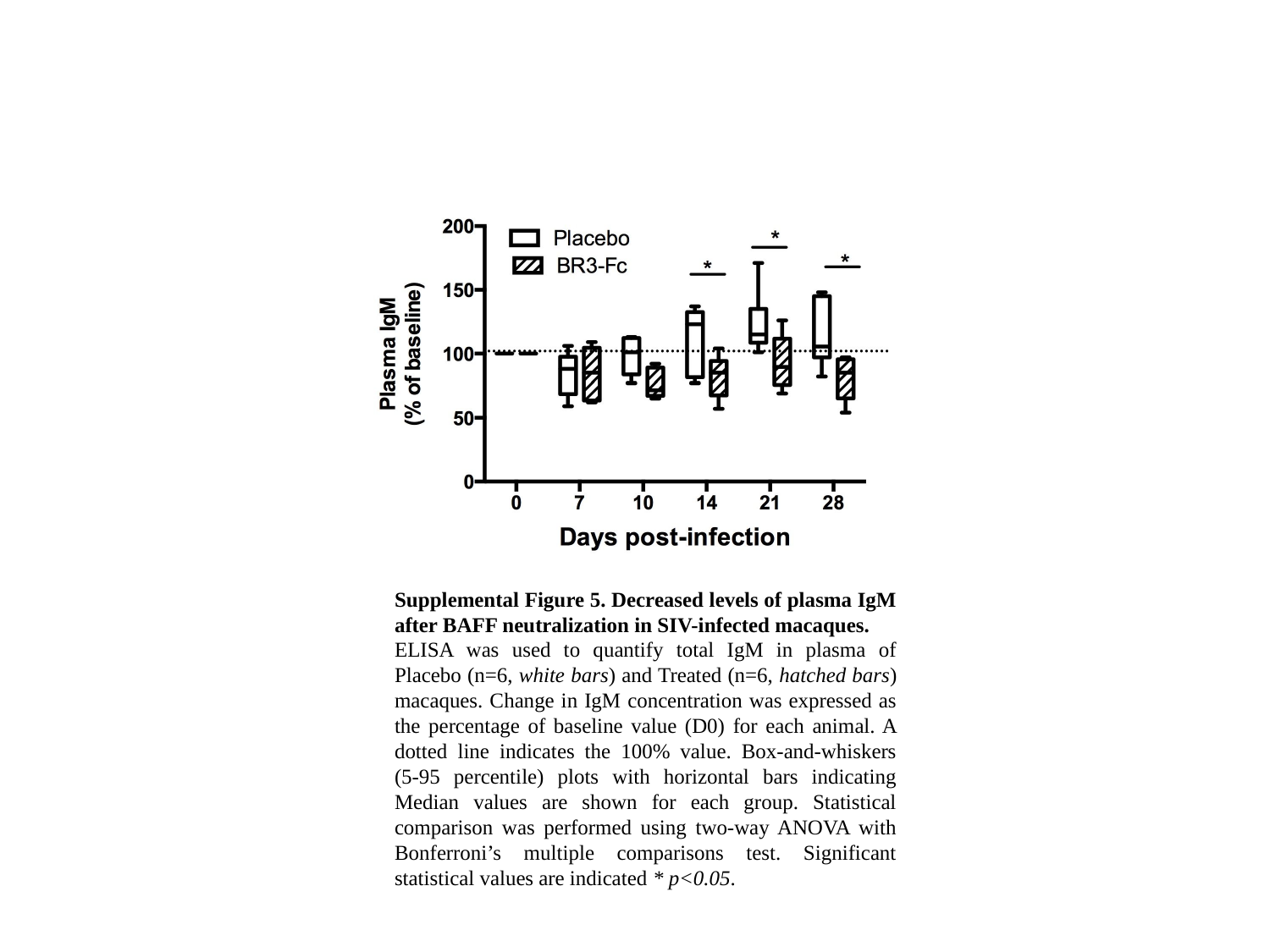

Supplemental Figure 5. Decreased levels of plasma IgM after BAFF neutralization in SIV-infected macaques.
ELISA was used to quantify total IgM in plasma of Placebo (n=6, white bars) and Treated (n=6, hatched bars) macaques. Change in IgM concentration was expressed as the percentage of baseline value (D0) for each animal. A dotted line indicates the 100% value. Box-and-whiskers (5-95 percentile) plots with horizontal bars indicating Median values are shown for each group. Statistical comparison was performed using two-way ANOVA with Bonferroni’s multiple comparisons test. Significant statistical values are indicated * p<0.05.
