# Supplementary material for: Impact of BAFF Blockade on Inflammation, Germinal Center Reaction and Effector B-Cells During Acute SIV Infection
Source: Front Immunol. 2020 Feb 28;11:252. doi: 10.3389/fimmu.2020.00252 (PMC7061218; doi:10.3389/fimmu.2020.00252)
Supplement: Supplementary file 12 [file Presentation_6.pptx]

## Slide 1
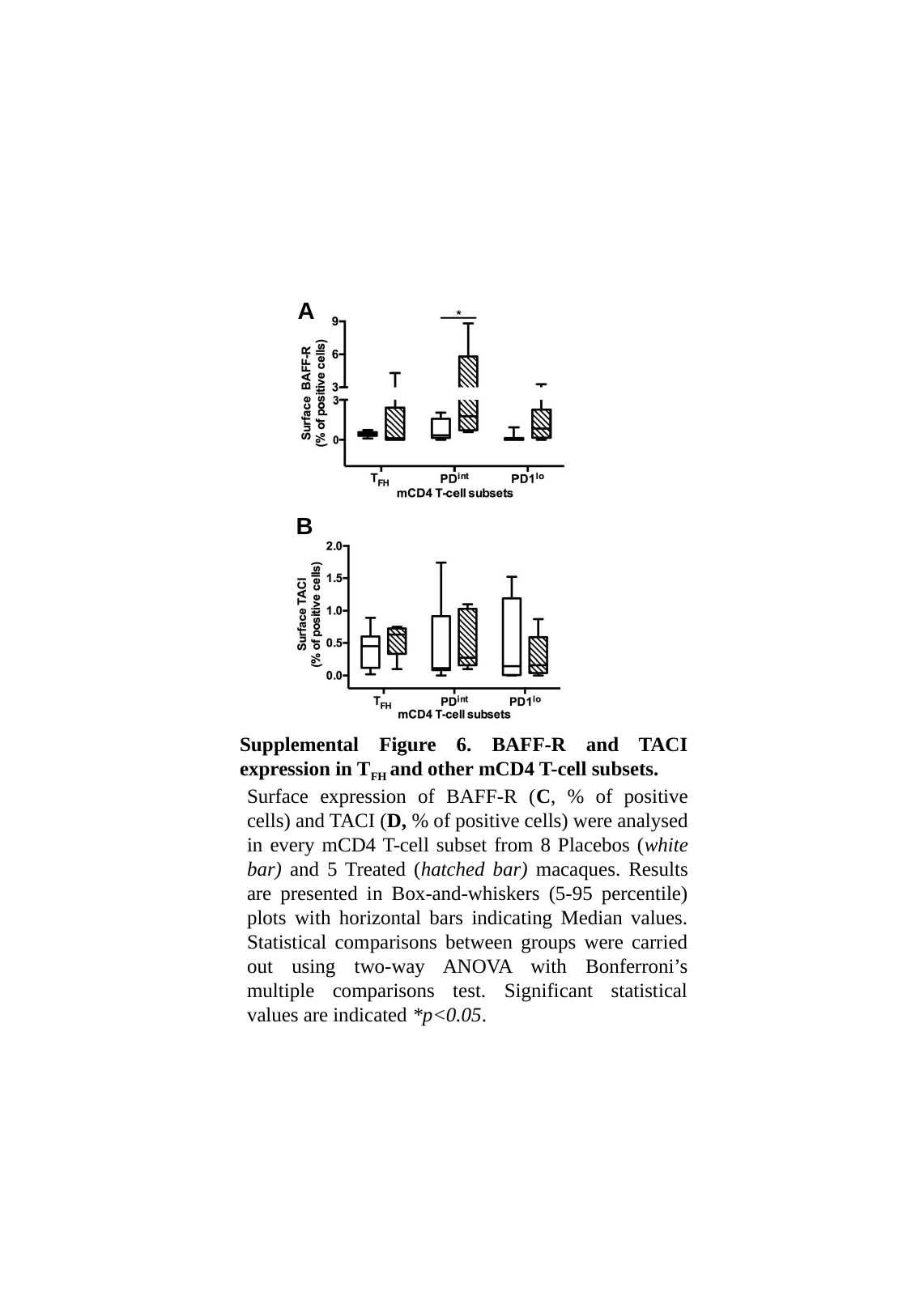

A
B
Supplemental Figure 6. BAFF-R and TACI expression in TFH and other mCD4 T-cell subsets.
Surface expression of BAFF-R (C, % of positive cells) and TACI (D, % of positive cells) were analysed in every mCD4 T-cell subset from 8 Placebos (white bar) and 5 Treated (hatched bar) macaques. Results are presented in Box-and-whiskers (5-95 percentile) plots with horizontal bars indicating Median values. Statistical comparisons between groups were carried out using two-way ANOVA with Bonferroni’s multiple comparisons test. Significant statistical values are indicated *p<0.05.
